# Digital Health Programs to Reduce Readmissions in Coronary Artery Disease: A Systematic Review and Meta-Analysis

**DOI:** 10.1016/j.jacadv.2023.100591

**Published:** 2023-09-07

**Authors:** Justin Braver, Thomas H. Marwick, Brian Oldenburg, Ayuba Issaka, Melinda J. Carrington

**Affiliations:** a Baker Department of Cardiometabolic Health, Faculty of Medicine, Dentistry and Health Sciences, The University of Melbourne, Victoria, Australia; b Pre-Clinical Disease and Prevention Unit, Baker Heart and Diabetes Institute, Victoria, Australia; c Non-Communicable Diseases and Implementation Science Unit, Baker Heart and Diabetes Institute, Victoria, Australia; d School of Psychology and Public Health, La Trobe University, Melbourne, Australia

**Keywords:** cardiac rehabilitation, coronary artery disease, digital health, disease management, health technology, mHealth

## Abstract

**Background:**

The use of mobile health (mHealth, wireless communication devices, and/or software technologies) in health care delivery has increased rapidly in recent years. Their integration into disease management programs (DMPs) has tremendous potential to improve outcomes for patients with coronary artery disease (CAD), yet a more robust evaluation of the evidence is required.

**Objectives:**

The purpose of this study was to undertake a systematic review and meta-analysis of mHealth-enabled DMPs to determine their effectiveness in reducing readmissions and mortality in patients with CAD.

**Methods:**

We systematically searched English language studies from January 1, 2007, to August 3, 2021, in multiple databases. Studies comparing mHealth-enabled DMPs with standard DMPs without mHealth were included if they had a minimum 30-day follow-up for at least one of all-cause or cardiovascular-related mortality, readmissions, or major adverse cardiovascular events.

**Results:**

Of the 3,411 references from our search, 155 full-text studies were assessed for eligibility, and data were extracted from 18 publications. Pooled findings for all-cause readmissions (10 studies, n = 1,514) and cardiac-related readmissions (9 studies, n = 1,009) indicated that mHealth-enabled DMPs reduced all-cause (RR: 0.68; 95% CI: 0.50-0.91) and cardiac-related hospitalizations (RR: 0.55; 95% CI: 0.44-0.68) and emergency department visits (RR: 0.37; 95% CI: 0.26-0.54) compared to DMPs without mHealth. There was no significant reduction for mortality outcomes (RR: 1.72; 95% CI: 0.64-4.64) or major adverse cardiovascular events (RR: 0.68; 95% CI: 0.40-1.15).

**Conclusions:**

DMPs integrated with mHealth should be considered an effective intervention for better outcomes in patients with CAD.

A concerning proportion of patients with coronary artery disease (CAD) have major risk factors,^1^ such that the residual lifetime risk for cardiovascular events and death could decrease if risk factor control and treatment improved.^2^ Optimal management following a cardiac event via secondary prevention cardiac rehabilitation (CR), and disease management programs (DMPs) is paramount to improve risk factors, enhance the prescription of cardio-protective medications, and reduce hospitalizations and mortality.^3^, ^4^, ^5^, ^6^, ^7^, ^8^, ^9^ However, despite unequivocal evidence for their effectiveness, CR programs are still underutilized with <50% of eligible patients referred worldwide.^1^^,^^10^ Consequently, cardiac readmission rates remain high and result in substantial costs. A major driver of these costs is hospitalization expenditure^11^^,^^12^ with the average cost of a 30-day readmission postacute myocardial infarction (AMI) costing approximately USD $15,000, with a cumulative cost of over USD $1 billion per year.^13^

The rapid use of mobile health (mHealth) technologies has produced strategies and modalities to overcome the historical challenges associated with traditional delivery of CR and DMPs. mHealth-delivered DMP interventions are newly recommended in guidelines,^14^ albeit based on lower-level quality evidence derived from a limited number of studies. An in-depth synthesis of the literature is required to keep abreast with the rapid boom in mHealth delivered secondary prevention cardiovascular disease (CVD) care.^15^ While previous systematic reviews in this field have shown improvements in clinical, behavioral, and lifestyle risk factors^16^, ^17^, ^18^, ^19^ (Supplemental Table 1), most prior systematic reviews, that investigated impact outcomes, involved mHealth interventions delivered via telephone.^20^, ^21^, ^22^ Telephone delivery is resource-intensive, time-consuming, and limits scalability. Less attention has been paid to the most up-to-date digital technologies, which enable a scalable and personalized service to numerous individuals. Further, the few systematic reviews that have attempted to address newer technologies^23^^,^^24^ included only a limited number of studies in their meta-analyses with mixed results. Therefore, the aim of this systematic review and meta-analysis was to develop evidence for the effectiveness of mHealth-enabled DMPs, excluding telephone only, on hospital readmissions and mortality in patients diagnosed with CAD.

## Methods

We conducted this systematic review in accordance with Preferred Reporting Items for Systematic Reviews and Meta-Analyses (PRISMA) guidelines^25^ and registered it with International Prospective Register of Systematic Reviews (PROSPERO) (CRD42022306749).

### Data sources and searches

MEDLINE, Embase database, the Cochrane Central Register of Controlled Trials, CINAHL, the Web of Science, and Scopus electronic databases were systematically searched for English language studies from January 1, 2007, to August 3, 2021. Grey literature was searched for additional papers. This start date was selected to coincide with the release of the Apple iPhone (the first internet-accessible smartphone with apps). The specific keywords, Medical Subject Heading terms, and search strategy are provided in Supplemental Table 2.

### Study selection

We used Covidence software for this systematic review.^26^ Two independent reviewers scanned the titles and abstracts of publications while a third reviewer adjudicated discrepancies. The full texts of selected studies were read in detail, and reasons for exclusion were recorded.

#### Inclusion criteria

Studies of patients who were discharged from hospital with CAD with a minimum of 30-days follow-up and at least 50 patients in the total sample that evaluated a DMP using mHealth compared with a standard DMP without mHealth were included. mHealth was defined as the use of wireless communication devices (mobile phones, smartphones, electronic tablets, and laptops) and/or software technology (apps, video and tele-conferencing, email, telemonitoring, social media, and SMS communication), excluding telephone-only interventions. A DMP is defined as a coordinated health care plan to help people manage their disease better. A DMP is the sum of activities that include some if not all of the following: health professional/nurse consultations, care coordination, regular follow-up, optimization of efficacious medications, education, psychological support, physical activity prescription, self-monitoring strategies (eg, blood pressure measurement), goal setting, and lifestyle/behavioral self-management strategies (eg, medication adherence and dietary intake). Studies were included if they contained at least one DMP component and reported outcomes for at least one of all-cause or cardiovascular mortality, all-cause or cardiovascular readmissions, or major adverse cardiovascular events (MACE).

#### Exclusion criteria

Studies were excluded if participants were not diagnosed with CAD or if they had heart failure. Interventions that did not involve mHealth, used the telephone only, or focused on a single behavior (eg, smoking cessation) were excluded.

### Data extraction and management

One reviewer extracted information about the study population, intervention and control/comparison group characteristics, and outcome data from each study using a predeveloped data extraction form. Ambiguities were resolved by discussion and consensus. Multiple publications of the same study were assessed for the provision of endpoint data and the most recent publication was chosen for inclusion.

### Assessment of risk bias and quality of the evidence

Risk of bias was assessed using the Cochrane Collaboration’s tool^27^ for randomized controlled trials and the ROBINS-I assessment tool^28^ for observational studies. Risk of bias plots were generated using ROBIS.^29^ GRADEpro GDT software^30^ was used to assess the quality of evidence for each outcome reported.

### Data synthesis and analysis

Analysis was performed using Review Manager (RevMan) version 5.3 software. We measured heterogeneity for each outcome across studies qualitatively by comparing study characteristics and quantitatively using the I^2^ test statistic. A meta-regression was performed to account for baseline differences between comparator groups for each outcome. Dichotomous variables were converted to log odds differences between comparator groups. Mean differences were used for continuous variables. We undertook subgroup analysis of: duration of DMP, length of follow-up, year of publication, patient characteristics, and intervention components (outlined in *Inclusion criteria*) to assess the effect of benefit from mHealth DMPs compared to standard DMPs.

We generated estimates of treatment effect using pooled RR with 95% CIs and random-effects models utilizing Mantel-Haenszel methods for combining results across studies. Data were pooled and displayed in forest plots. Hypothesis testing was set at the 2-tailed 0.05 level. The funnel plot and Egger test were used to examine publication bias (Supplemental Figure 1).^31^

## Results

**Figure 1:**
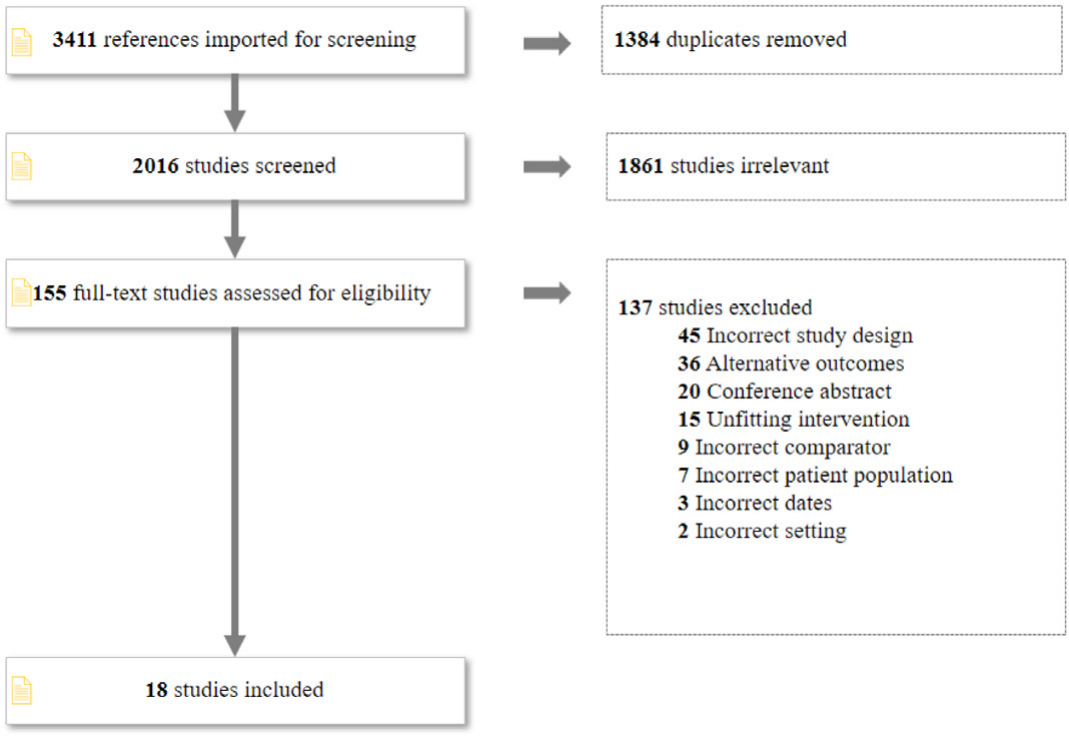
**Study Selection** The search yielded 3,411 references, 155 full text studies were assessed for eligibility and data were extracted from 18 publications (16 RCTs and 2 non-RCTs). RCT = randomized controlled trial.

As shown in Figure 1, our initial search yielded 3,411 references. After the removal of 1,384 duplicates, 2,016 were reviewed for title and abstract eligibility. Of these, we assessed 155 full-text studies to include a total of 18 publications in the systematic review.

### Publication characteristics

**Figure 2:**
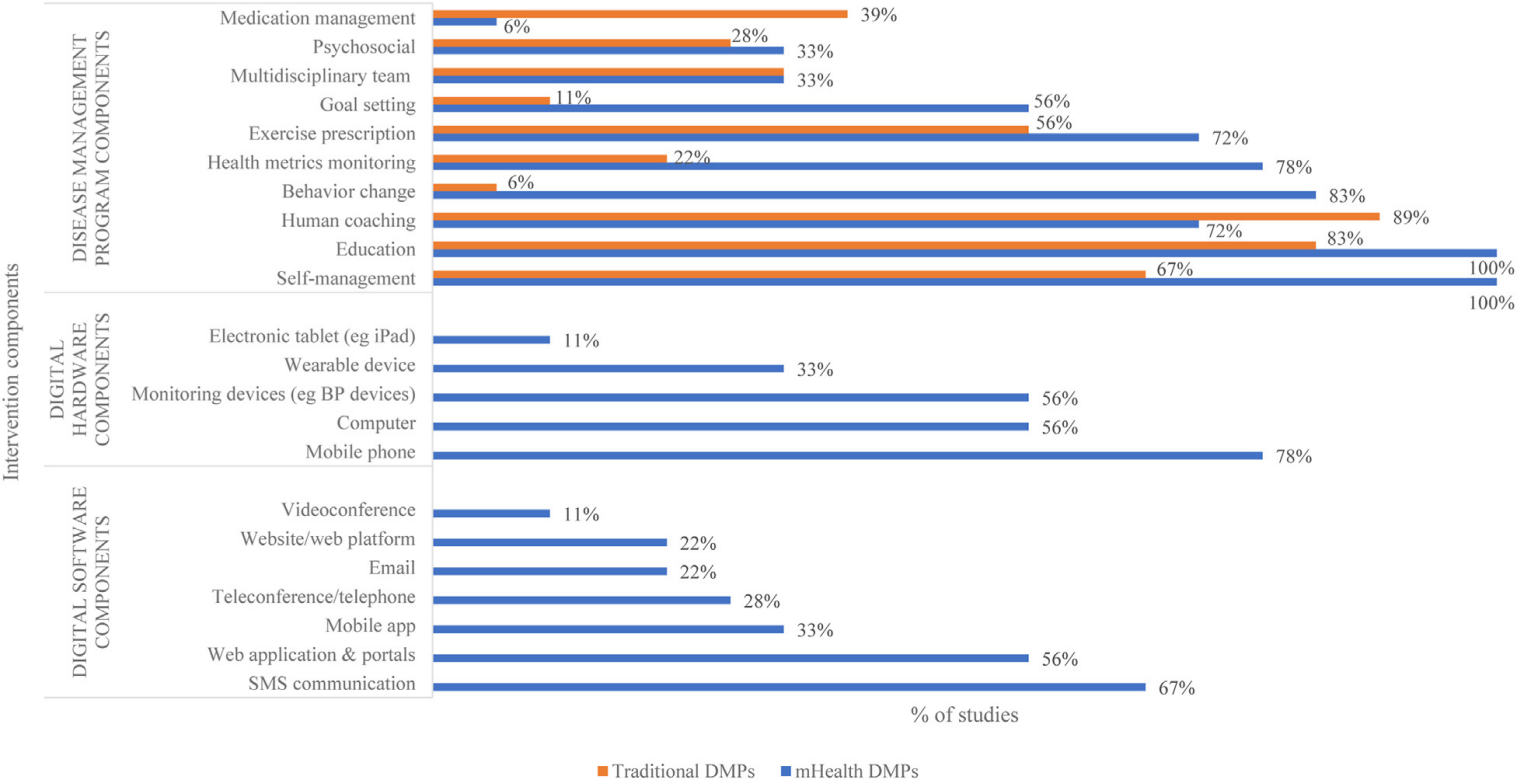
**Intervention Characteristics mHealth DMPs vs Traditional DMPs** Text messages (SMS) were the most common mHealth component^32^, ^33^, ^34^, ^35^, ^36^, ^37^, ^38^, ^39^, ^40^, ^41^, ^42^, ^43^ followed by web applications^34^^,^^37^^,^^38^^,^^41^^,^^42^^,^^44^, ^45^, ^46^, ^47^, ^48^ and mobile apps.^34^^,^^35^^,^^37^^,^^39^^,^^43^^,^^48^ Mobile phones were the most common hardware component.^32^, ^33^, ^34^, ^35^, ^36^, ^37^, ^38^, ^39^, ^40^, ^41^, ^42^, ^43^^,^^45^^,^^48^ DMP = disease management program; mHealth = mobile health.

Table 1, Figure 2, and Supplemental Table 3 outline details of the study and intervention characteristics. In summary, included studies were published between 2008 and 2021, and the majority were RCTs (89%). Most studies were conducted in Europe,^32^, ^33^, ^34^^,^^44^^,^^45^ North America and Canada,^35^^,^^46^, ^47^, ^48^, ^49^ and Australasia.^36^, ^37^, ^38^, ^39^ Most studies (13 publications) reported follow-up within 6 months,^32^^,^^34^, ^35^, ^36^, ^37^, ^38^, ^39^, ^40^, ^41^, ^42^, ^43^^,^^46^^,^^48^ 4 reported 12-month follow-up,^44^^,^^45^^,^^47^^,^^49^ and one study reported 24-month follow-up.^33^

**Table 1:** Publication and mHealth Intervention Characteristics

First Author	Year of Publication	Study Country	Study Design	Length of Follow-Up	Duration of DMP	Setting	Software(s)	Digital Hardware(s)	DMP Components
Bae	2021	Korea	RCT	6 mo	24 wk	Home & center based	1-way sms (delivered via message-sending program) & supporting info website	Mobile phone & computer	Education; meds mgmt; exercise prescription; self-management; psychosocial support; behavior change; goal setting
Chow	2015	Australia	RCT	6 mo	24 wk	Home	1-way sms delivered via automated computerized message management system	Mobile phone	Education; meds mgmt; exercise prescription; self-management; psychosocial support; behavior change
Frederix	2015	Belgium	RCT	4.5 mo	18 wk	Home & hybrid	1-way sms; email & web application/web portal; telemonitoring	Mobile phone; motion sensor/wearable device & computer	Health professional consultations; education; meds mgmt; exercise prescription; metrics monitoring; self-management; psychosocial support; behavior change
Frederix	2017	Belgium	RCT	24 mo	24 wk	Home, center based & hybrid	1-way sms; email & web application/secure webpage (telemonitoring)	Mobile phone; motion sensor/wearable device (Yorbody accelerometer) & computer	Health professional consultations; education; exercise prescription; metrics monitoring; self-management; goal setting; multidisciplinary team
Khonsari	2015	Malaysia	RCT	2 mo	8 wk	Home	1-way sms; automated sms-based reminder system & telephone calls	Mobile phone & computer	Education; meds mgmt; self-management; behavior change
Khonsari	2020	Iran	Feasibility RCT	3 mo	12 wk	Home	1-way sms & automated sms-based reminder system	Mobile phone & computer	Education; meds mgmt; self-management; behavior change
Kraal	2017	the Netherlands	RCT	12 mo	12 wk	Home, center based & hybrid	Telemonitoring with web application (Garmin Connect); patient facing + provider facing web application & telephone calls;	Heart rate monitor with a chest strap (Garmin FR70); computer & telephone	Health professional consultations; education; exercise prescription; metrics monitoring; self-management; behavior change; multidisciplinary team
Maddison	2019	New Zealand	RCT	6 mo	12 wk	Home, center based & hybrid	Mobile application; 1- & 2-way sms; web application (provider facing); telemonitoring; & tele-conferencing	Mobile phone (smartphone) & smartphone and chest-worn wearable sensor	Health professional consultations; education; exercise prescription; metrics monitoring; self-management; psychosocial support; behavior change; goal setting; multidisciplinary team
Mcelroy	2016	USA	Non-randomized Prospective study	1 mo	4 wk	Home & center based	Telemonitoring; web application; digital questionnaires; video conferencing; telephone	Digital health kits: tablet linked to a Bluetooth-enabled pulse oximeter, heart rate monitor, blood pressure cuff, and weight scale & telephone	Health professional consultations; education; meds mgmt; exercise prescription; metrics monitoring; self-management; multidisciplinary team
Pakrad	2021	Iran	RCT	6 mo	16 wk	Home, center based & hybrid	Mobile application & 2-way communication	Mobile phone	Health professional consultations; education; meds mgmt; exercise prescription; metrics monitoring; self-management; behavior change; goal setting
Pfaeffli	2015	New Zealand	RCT	6 mo	24 wk	Home & center based	2-way sms; supporting website/web portal; telemonitoring; participant blog	Mobile phone; computer & pedometer	Health professional consultations; education; meds mgmt; exercise prescription; metrics monitoring; self-management; psychosocial support; behavior change; goal setting
Reid	2012	Canada	RCT	12 mo	24 wk	Home	Web portal/secure website; email & online tutorials	Computer & pedometer	Health professional consultations; education; exercise prescription; metrics monitoring; self-management; behavior change; goal setting
Riegel	2020	USA	RCT	3 mo	12 wk	Home	Mobile application & electronic messages	Mobile phone & electronic pill device	Education; meds mgmt; metrics monitoring; self-management; behavior change
Snoek	2019	the Netherlands	RCT	12 mo	24 wk	Home & center based	Telemonitoring; web application/web portal & telephone	Mobile phone (Samsung Galaxy); Bluetooth-connected heart rate belt & computer	Health professional consultations; education; exercise prescription; metrics monitoring; self-management; behavior change; goal setting; multidisciplinary team
Widmer	2017	USA	RCT	6 mo	12 wk	Home, center based & hybrid	Mobile application; email; web-based portal; telemonitoring; online messaging;	Mobile phone & computer	Health professional consultations; education; meds mgmt; exercise prescription; metrics monitoring; self-management; behavior change; goal setting
Wolf	2016	Sweden	Sub study of a RCT	6 mo	8 wk-6 mo	Home & hybrid	Mobile application; web application/webpage & 2-way messaging/chat function	Mobile phone & computer	Health professional consultations; education; metrics monitoring; self-management; behavior change; goal setting; multidisciplinary team
Woodend	2008	Canada	RCT	12 mo	12 wk	Home	Video conferencing & telemonitoring	Home monitoring equipment; electronic weigh scales; blood pressure, and electrocardiogram machines	Health professional consultations; education; metrics monitoring; self-management
Yudi	2021	Australia	RCT	2 mo	8 wk	Home, center based & hybrid	Mobile application & personalized feedback	Mobile phone	Health professional consultations; education; meds mgmt; exercise prescription; metrics monitoring; self-management; psychosocial support; behavior change; goal setting; multidisciplinary team

DMP = disease management program; RCT = randomized controlled trial.

The intervention duration of most DMPs lasted 12 to 24 weeks.^32^^,^^33^^,^^35^, ^36^, ^37^, ^38^^,^^40^^,^^41^^,^^43^, ^44^, ^45^^,^^47^, ^48^, ^49^ All mHealth-enabled DMPs were delivered remotely, yet in 9 studies, some aspects of the model of care were undertaken in a face-to-face setting.^32^, ^33^, ^34^^,^^37^, ^38^, ^39^^,^^43^^,^^44^^,^^48^ The most common digital component reported in 12 studies were text messages (SMS).^32^, ^33^, ^34^, ^35^, ^36^, ^37^, ^38^, ^39^, ^40^, ^41^, ^42^, ^43^ Ten studies reported web applications and/or provider dashboards entailing online portals for reviewing patient-generated data from remote monitoring.^34^^,^^37^^,^^38^^,^^41^^,^^42^^,^^44^, ^45^, ^46^, ^47^, ^48^ Mobile apps were used in one-third of studies.^34^^,^^35^^,^^37^^,^^39^^,^^43^^,^^48^ Mobile phones were the most common hardware component used in 14 studies.^32^, ^33^, ^34^, ^35^, ^36^, ^37^, ^38^, ^39^, ^40^, ^41^, ^42^, ^43^^,^^45^^,^^48^ Within the mHealth-enabled DMP group, all studies included self-management strategies and education; 13 included health professional coaching^32^, ^33^, ^34^^,^^37^, ^38^, ^39^^,^^43^, ^44^, ^45^, ^46^, ^47^, ^48^, ^49^ and exercise prescription^32^^,^^33^^,^^36^, ^37^, ^38^, ^39^, ^40^^,^^43^, ^44^, ^45^, ^46^, ^47^, ^48^ and 14 studies incorporated monitoring of health metrics.^32^, ^33^, ^34^, ^35^^,^^37^, ^38^, ^39^^,^^43^, ^44^, ^45^, ^46^, ^47^, ^48^, ^49^

CAD subvariants could be delineated in 9 studies.^34^^,^^37^, ^38^, ^39^, ^40^^,^^42^^,^^44^^,^^47^^,^^49^ Of these, patients were hospitalized with acute CAD after an AMI (58% intervention and 61% control) or unstable angina (42% intervention and 39% control), and the most common treatment was with percutaneous coronary intervention (84% intervention and 85% control) compared with coronary artery bypass graft (32% intervention and 38% control). AMI type was reported in 3 papers^34^^,^^39^^,^^42^ as ST-segment elevation myocardial infarction (34% intervention and 28% control) and non-ST-segment elevation myocardial infarction (mean 34% intervention and 46% control). Overall, 3,818 patients were included ranging from 62 to 879 patients per study. The weighted average age of the intervention and control groups was 60.3 ± 1.3 years and 62.6 ± 1.15 years, respectively, and the majority were men (82% intervention and 80% control). Pooled baseline characteristics were similar for the mHealth DMP group and the DMP group alone, aside from a higher proportion of current smokers (24% vs 19%) and family history of CVD (53% vs 33%) in the mHealth DMP group (Supplemental Table 4).

### Primary outcome analysis

**Figure 3:**
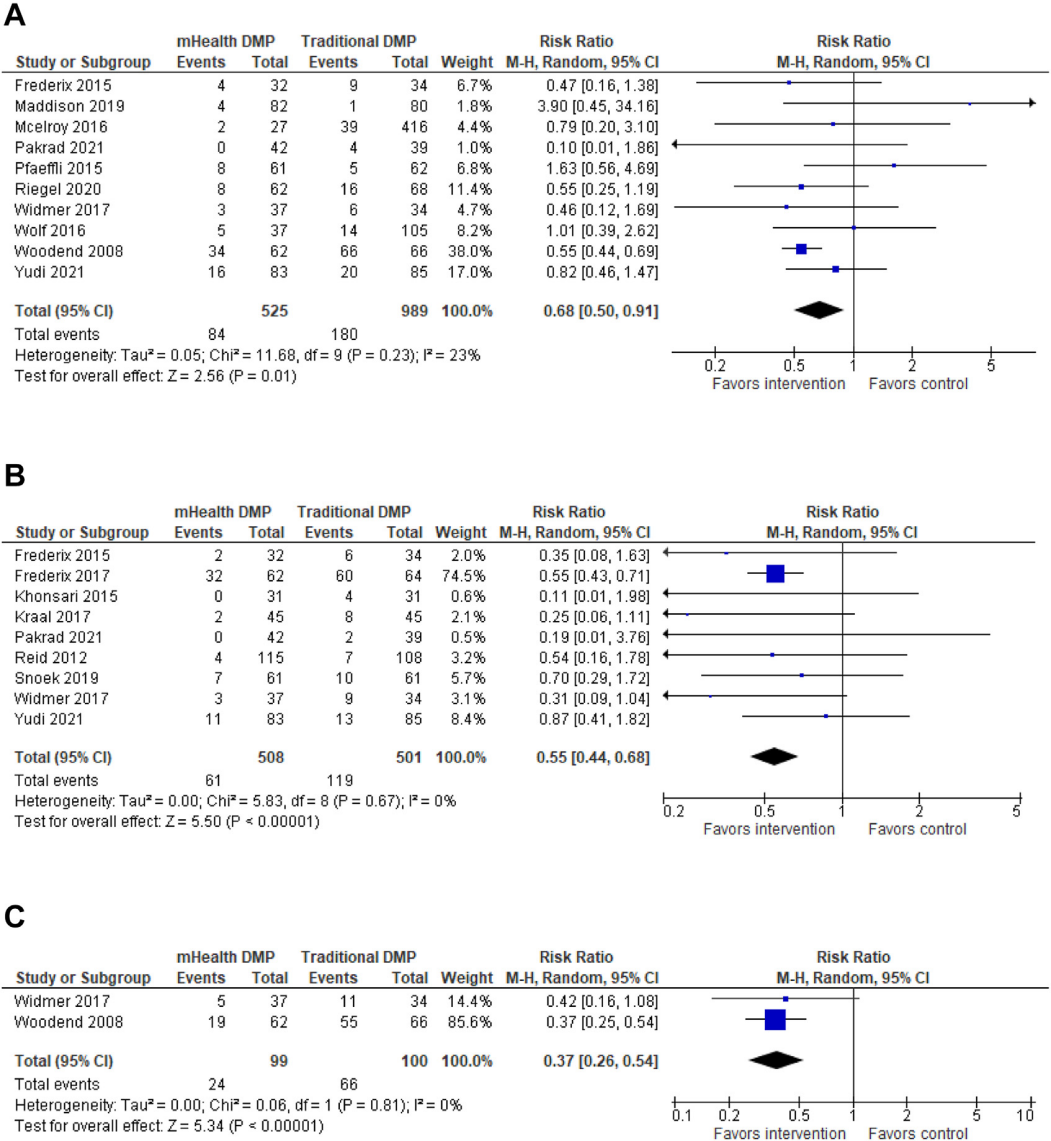
**Primary Outcome Analysis** (A) All-cause readmissions. Risk for all-cause readmission (n = 1,514) is reduced by 32% in the mHealth DMP group compared to DMPs without mHealth. (B) Cardiac-related readmissions. Risk for cardiovascular related readmission is reduced by 45% in the mHealth DMP group compared to DMPs without mHealth. (C) ED visits. The risk for emergency department visits is reduced by 63% in favor of the mHealth-enabled DMP group compared to DMPs without mHealth. DMP = disease management program; ED = emergency department.

**Figure 4:**
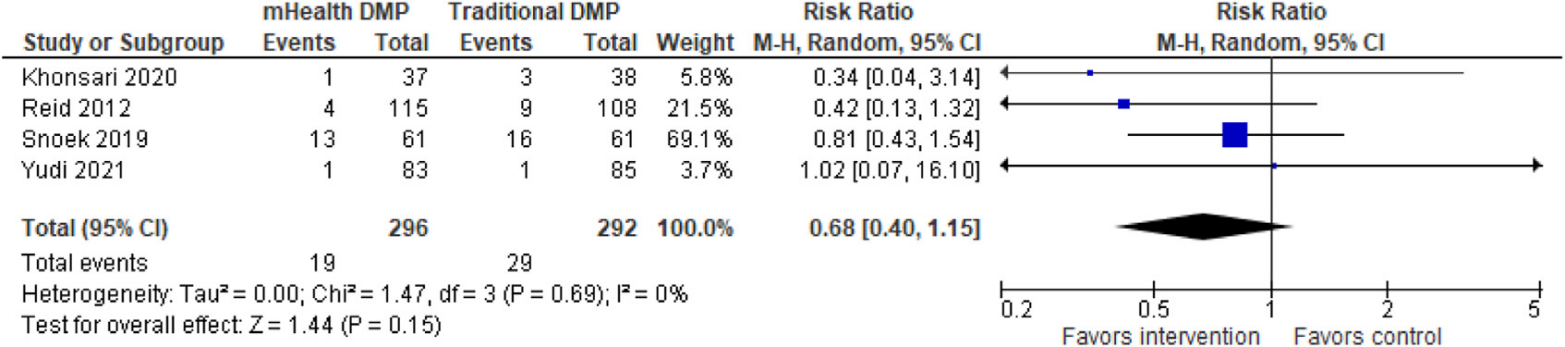
**Primary Outcome Analysis: MACE** There was no significant effect of mHealth DMPs on MACE compared to DMPs without mHealth. DMP = disease management program; MACE = major adverse cardiac event; mHealth = mobile health.

**Figure 5:**
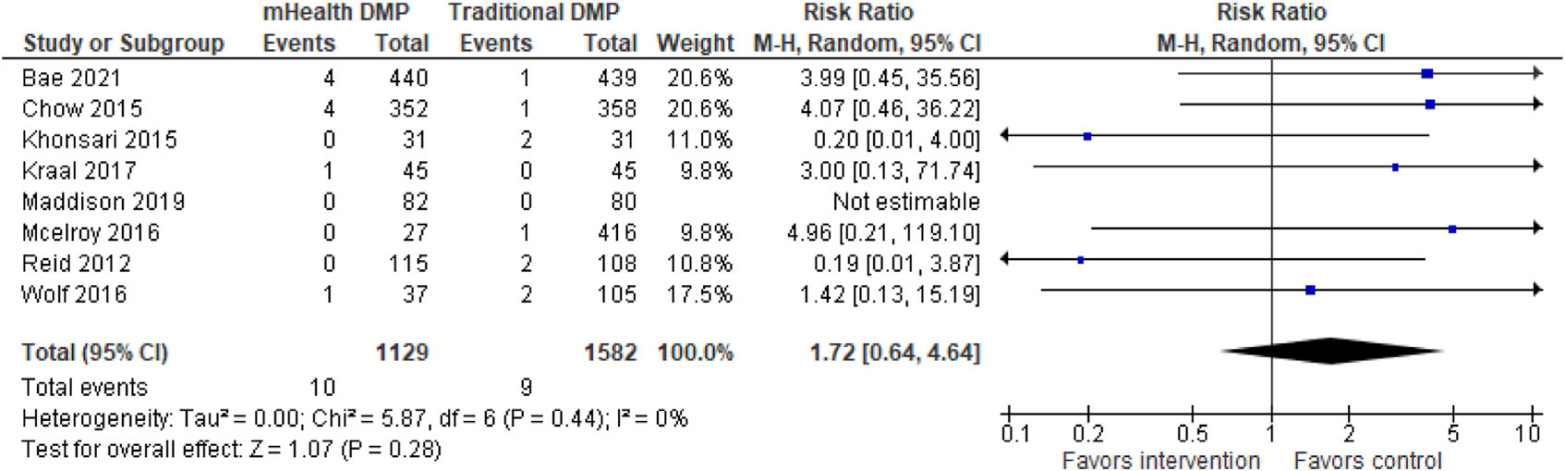
**Primary Outcome Analysis: All-Cause Mortality** There was no risk reduction for all-cause mortality in the mHealth-enabled DMP group compared to DMPs without mHealth. DMP = disease management program; mHealth = mobile health.

The results for dichotomous primary outcome data are shown in separate forest plots for hospital encounters (Figure 3), MACE (Figure 4), and mortality (Figure 5).

#### Readmissions

Ten studies assessed all-cause readmissions,^32^^,^^34^^,^^35^^,^^37^, ^38^, ^39^^,^^43^^,^^46^^,^^48^^,^^49^ and 9 studies assessed cardiovascular readmissions.^32^^,^^33^^,^^39^^,^^42^, ^43^, ^44^, ^45^^,^^47^^,^^48^ Pooled analysis showed that risk for all-cause readmission (n = 1,514) (Figure 3A) was reduced by 32% (RR: 0.68; 95% CI: 0.50-0.91) and cardiovascular readmissions (n = 1,009) (Figure 3B) by 45% (RR: 0.55; 95% CI: 0.44-0.68) in the mHealth-enabled DMP group compared to the DMP alone group. There was no evidence of competing risk analysis whereby mortality may lead to a reduction in readmission given there were a total of 4 deaths from 1,514 patients included in all-cause readmission analysis and 5 deaths from 1,009 patients included in cardiac-related readmission analysis.

#### ED visits and MACE

From 2 studies^48^^,^^49^ (n = 199) reporting emergency department (ED) visits (Figure 3C), risk was reduced by 63% in favor of the mHealth-enabled DMP group (RR: 0.37; 95% CI: 0.26-0.54) compared with the traditional DMP control group. Four studies^39^^,^^41^^,^^45^^,^^47^ (n = 588) (Figure 4) assessed MACE, and there was no significant effect of a mHealth DMP relative to a standard DMP (RR: 0.68; 95% CI: 0.40-1.15).

#### Mortality

Eight studies^34^^,^^36^^,^^37^^,^^40^^,^^42^^,^^44^^,^^46^^,^^47^ (n = 2,711) assessed all-cause mortality. As shown in Figure 5, there was no risk reduction for all-cause mortality (RR: 1.72; 95% CI: 0.64-4.64) in the mHealth-enabled DMP group compared with the traditional DMP alone group. There were no included studies reporting cardiac-related deaths.

There was no evidence of statistical heterogeneity in the sets of studies for all primary outcomes aside from a small amount for all-cause readmissions (I^2^ = 23%). Stratified meta-regression revealed no baseline differences between comparator groups for any primary outcome (Supplemental Table 5). Subgroup analysis using pooled data revealed no significant group differences (Supplemental Table 6). There were no group differences after removing the 2 observational studies.

### Risk of bias and grade assessment

The overall risk of bias across domains for each study was judged to be low or unclear (Supplemental Figure 2). The GRADE quality of evidence for each outcome was assessed as moderate for all-cause readmissions, high for cardiac-related readmissions and ED visits, low for MACE and very low for all-cause mortality (Table 2, Supplemental Table 7). There was no evidence of funnel plot asymmetry or significant Egger tests (Supplemental Figure 1), and thus no evidence of publication bias.

**Table 2:** Summary Findings of Grade Quality Assessment

	Anticipated Absolute Effects^a^ (95% CI)		Relative Effect (95% CI)	No. of Participants (Studies)	Certainty of the Evidence (GRADE)
	Risk With Traditional DMPs Alone	Risk With mHealth DMPs			
All-cause readmissions	18 per 100	12 per 100 (9-17)	RR: 0.68 (0.50-0.91)	1,514 (8 RCTs and 2 non-RCTs)	⨁⨁⨁◯ Moderate
Cardiac-related readmissions	24 per 100	13 per 100 (10-16)	RR: 0.55 (0.44-0.68)	1,009 (9 RCTs)	⨁⨁⨁⨁ High
ED visits	66 per 100	24 per 100 (17-36)	RR: 0.37 (0.26-0.54)	199 (2 RCTs)	⨁⨁⨁⨁ High
MACE	10 per 100	7 per 100 (4-11)	RR: 0.68 (0.40-1.15)	588 (4 RCTs)	⨁⨁◯◯ Low
All-cause mortality	1 per 100	1 per 100 (0-3)	RR: 1.72 (0.64-4.64)	2,711 (6 RCTs and 2 non-RCTs)	⨁◯◯◯ Very low

GRADE Working Group grades of evidence. High certainty: we are very confident that the true effect lies close to that of the estimate of the effect. Moderate certainty: we are moderately confident in the effect estimate; the true effect is likely to be close to the estimate of the effect, but there is a possibility that it is substantially different. Low certainty: our confidence in the effect estimate is limited; the true effect may be substantially different from the estimate of the effect. Very low certainty: we have very little confidence in the effect estimate: the true effect is likely to be substantially different from the estimate of effect.

GRADEpro GDT software^30^ was used to assess the quality of evidence for each outcome reported. The GRADE quality of evidence for each outcome was assessed as moderate for all-cause readmissions, high for cardiac-related readmissions and ED visits, low for MACE, and very low for all-cause mortality.

ED = emergency department; MACE = major adverse cardiac event; RR = risk ratio.

a The risk in the intervention group (and its 95% CI) is based on the assumed risk in the comparison group and the relative effect of the intervention (and its 95% CI).

## Discussion

In this systematic review and meta-analysis, mHealth-enabled DMPs for patients with CAD were effective interventions for reducing hospital readmissions and visits to ED. However, there was no greater benefit for mHealth-enabled DMPs on mortality or MACE outcomes (Central Illustration). Findings did not vary across any patient, intervention, or study characteristics. Our results update the evidence for the effectiveness of mHealth-enabled secondary prevention DMPs by including more studies that assessed impact outcomes (hospitalizations, ED visits, MACE, and mortality) and using only the latest digital technologies over and above telephone communication.

**Central Illustration:**
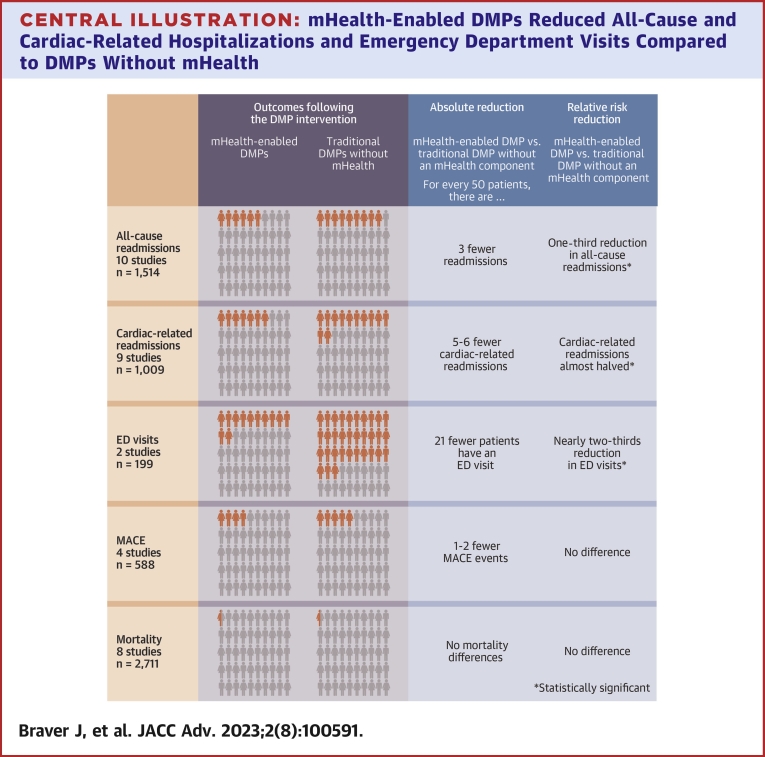
**mHealth-Enabled DMPs Reduced All-Cause and Cardiac-Related Hospitalizations and Emergency Department Visits Compared to DMPs Without mHealth** There was no significant reduction for mortality outcomes or MACE. DMP = disease management program; MACE = major adverse cardiac event; mHealth = mobile health.

Our findings indicated a 32% reduction in the relative risk of rehospitalization for any cause and a 45% relative risk reduction in cardiovascular-related rehospitalizations in mHealth-enabled DMP patients compared with patients who undertook a traditional DMP. This contrasts with a prior systematic review that used text messaging or mobile phone app interventions^23^ but aligns with others incorporating telephone call interventions, which showed a reduction of between 38% and 44% in all-cause rehospitalizations compared with standard postdischarge secondary prevention care.^20^^,^^22^ Overall, mHealth DMPs are effective and complement existing telephone-based interventions. mHealth-enabled DMPs support the scalability of existing models of care, enhance patient motivation and adherence, and achieve effective results.^50^ They also create cost efficiencies for health care delivery by reducing clinician and health system burden.^51^^,^^52^ Hence, rather than replacing the entire traditional model of care with a digital solution, digitally integrated models may provide disease management strategies in a more engaging, accessible, and scalable manner.^53^

It appears that the beneficial effects of novel mHealth DMPs are due to the sum of their parts. The evidence suggests that there is no one specific component that is the key but rather a combination of factors working together to improve provider-patient communication and enhance patient-centered care. These factors combined enhance engagement, adherence, and subsequent outcomes.^24^^,^^50^^,^^54^

Our study provides evidence for the effectiveness of mHealth interventions (incorporating digital technologies) for reducing readmissions and ED visits in patients with CAD. These tech-integrated models of DMPs provide unique opportunities for providers and health systems to interact directly with patients’ contemporary lifestyles, delivering more personalized patient-centered care. Rapid technological advancement, improved user experience, and positive consumer acceptance and adoption (from patients and providers)^55^^,^^56^ have enhanced engagement and adherence^16^^,^^57^ to prevention programs and may explain the added benefit of mHealth-enabled DMPs over and above traditional DMPs without mHealth.

Despite almost all earlier systemic reviews showing significant improvements in clinical, behavioral and lifestyle risk factors when comparing digital technology interventions with traditional DMPs or usual care,^16^, ^17^, ^18^, ^19^ previous studies have not investigated the impact of mHealth interventions on readmission and mortality outcomes using emerging digital technologies and devoid of telephone only interventions (Supplemental Table 1). There is heterogeneity between DMP interventions such that more tangible benefits might be realized from improved self-care/behavior change strategies and symptom awareness. These patient-focused behaviors may result in effective risk factor reduction and minimize exacerbation of CVD (including the onset of other events) rather than reduce mortality.

While our results provide evidence for mHealth interventions in lowering readmission risk, a consistent finding is that there is no evidence for reducing mortality.^17^^,^^20^^,^^23^^,^^24^ This may be due to comparator groups^20^ (either standard care, traditional DMP or cardiac rehabilitation) receiving close to optimal care (Supplemental Tables 8 and 9) or study populations being at low risk of mortality.^17^ Given the large heterogeneity between DMP interventions, there is also difficulty in assessing the overall impact on survival rates and health outcomes. Importantly, many studies include relatively short follow-up periods, which may be too short to detect longer-term impacts on mortality.

The results of this systematic review support wider implementation of mHealth-enabled DMPs in secondary prevention settings and should be made accessible to all CAD patients to choose their preferred DMP type and setting. In doing so, one needs to consider the implication for vulnerable or disadvantaged patients. We must ensure to continue to innovate and drive rapid translational research in digital health, but at the same time, consideration must be placed not to exacerbate health inequalities.^58^ Referral to CVD DMPs is often inequitable, with lower referral rates for older adults, women, under-represented minority groups, lower socioeconomic status populations, and those living in remote and regional areas.^59^, ^60^, ^61^ This is notable because many of these populations have greater rates of CVD compounded by less access to care.^62^ Additional research is needed to strengthen equitable access to digital health-based DMPs for these key populations^58^ and investigate the factors that are important for implementation of mHealth-enabled DMPs in real-world settings, particularly in low- and middle-income countries.

### Strengths and limitations

This systematic review and meta-analysis provides evidence for the effectiveness of the most contemporary mHealth-enabled DMPs on readmission outcomes. There are a few limitations to our study. Firstly, the limited availability of mortality outcomes with a relatively short follow-up period made it challenging to assess the intervention’s effect on mortality. Secondly, while we extracted all available data in each publication, adjudication of cardiovascular events that constitute a cardiovascular readmission may vary between studies, and similarly, noncardiovascular-related readmissions may not have been included among all studies. Finally, most studies included were conducted in high-income countries, yet more than 75% of CVD deaths take place in low- and middle-income countries.^63^ Hence, caution is required with regards to generalizability of the findings in these less represented populations.

## Conclusions

In this contemporary systematic review and meta-analysis, mHealth-integration into DMPs was an effective intervention for reducing hospital readmissions and visits to ED. DMPs supported by mHealth should be considered for improving outcomes in patients with CAD.

PERSPECTIVES **COMPETENCY IN PATIENT CARE:** Traditional secondary prevention DMPs improve risk factors and reduce hospitalizations. However, they are underutilized worldwide. Telephone delivered DMPs are also effective in improving health outcomes, but they are less scalable and increase clinician burden. The results of this study provide updated evidence for the effectiveness of mHealth interventions for reducing readmissions and ED visits in patients with CAD. Providing patients with the choice, access, and control of their care via mHealth-enabled DMPs should be considered when offering secondary prevention care to patients.

**TRANSLATIONAL OUTLOOK:** Alternative and cost-effective models of DMPs are required to increase access and engagement to care and reduce preventable and costly readmissions. Further research is required to understand the factors that are important for implementation of mHealth-enabled DMPs, particularly in disadvantaged populations that are at higher risk for CAD.

## Funding support and author disclosures

Mr Braver has received a postgraduate research scholarship from the University of Melbourne and the Baker Institute. Associate Professor Carrington has received an endowed fellowship in the Cardiology Center of Excellence from Filippo and Maria Casella. Supported in part by 10.13039/501100000925National Health and Medical Research Council (NHMRC) funding for the Center of Research Excellence in Digital Technology to Transform Chronic Disease Outcomes (APP 1170937) awarded to Dr Oldenburg. All other authors have reported that they have no relationships relevant to the contents of this paper to disclose.
