# Supplementary material for: Digital Health Programs to Reduce Readmissions in Coronary Artery Disease: A Systematic Review and Meta-Analysis
Source: JACC Adv. 2023 Sep 7;2(8):100591. doi: 10.1016/j.jacadv.2023.100591 (PMC11198697; doi:10.1016/j.jacadv.2023.100591)
Supplement: Supplemental Tables 1-9, and Supplemental Figures 1 and 2 [file mmc1.docx]

| Supplemental Table 1: Summary of previous systematic reviews and gap analysis | | | | | | | | | |
| --- | --- | --- | --- | --- | --- | --- | --- | --- | --- |
| **Systematic review** | **Description** | **Comparator** | **Outcomes** | | | | | | |
|  |  |  | **Process outcomes** | **Clinical & behavioural** | **All cause readmissions** | **Cardiac related readmissions** | **ED visits** | **MACE** | **All-cause mortality** |
| **Neubeck 2009(1)** | Telehealth interventions on risk factor modification in patients with CHD | Usual care |  | **✓** |  |  |  |  | **✓**  **11 studies** |
| **Widmer 2015(2)** | Any element of digital health intervention (DHI)(telemedicine, web-based strategies, email, mobile phones, mobile applications, text messaging, and monitoring sensors) and impact on CVD | Usual care following standard guidelines  Could involve non-DHI intervention (such as paper instructions or telephone calls)  No active intervention beyond usual care |  | **✓** | **✓**  **5 studies*** |  |  |  | **✓**  **5 studies*** |
| **Clark 2015(3)** | Critique the evidence for the effectiveness of alternative models of cardiac rehab | Traditional hospital-based approach  A model of care compared with another model  No comparison | **✓** | **✓** |  |  |  |  |  |
| **Huang 2015(4)** | Evaluation of the two forms of CR (telehealth intervention versus center-based CR) carried out within randomized controlled trials | Centre-based cardiac rehab |  | **✓** | Not specified | Not specified |  |  | **✓**  **8 studies** |
| **Gandhi 2017(5)** | Investigated the effect of mhealth technologies on outcomes in patients with CVD | Usual care (defined as all aspects of standard of care aside from the mhealth intervention | **✓** | **✓** | **✓**  **5** **studies** |  |  |  | **✓**  **2 studies** |
| **Hamilton 2018(6)** | mHealth interventions for CR and heart failure management for service and patient outcomes, cost effectiveness with a view to how mHealth could be utilized for rural, remote and Indigenous cardiac patients | No intervention  Traditional cardiac rehabilitation  usual care | **✓** | **✓** |  |  |  |  |  |
| **Jin 2019(7)** | To determine whether telehealth interventions can provide effective secondary prevention as an alternative or adjunct care compared with cardiac rehabilitation and/or usual care for patients with CHD | Usual care (routine care CHD)  Traditional cardiac rehabilitation | **✓** | **✓** | **✓**  7 studies | Not specified (grouped with all-cause) |  |  | **✓**  14 studies |
| **Su 2020(8)** | Assess the effects of eHealth cardiac rehab on behavioural, physiological and clinical outcomes | No interventions  Waiting lists  Usual care from services  Active comparison such as different level of eHealth or centre-based CR | **✓** | **✓** | **✓**  3 studies |  |  |  | **✓**  5 studies |
| **Wongvibulsin 2020(9)** | Digital interventions for cardiac rehab & identify the key components of CR that have been successfully addressed through digital interventions | n/a | **✓** | **✓** |  |  |  |  |  |
| **Subedi 2020(10)** | Evaluating the effectiveness of implementing cardiac telerehabilitation interventions at scale in routine clinical practice | n/a | **✓** |  |  |  |  |  |  |
| **Murphy 2020(11)** | The effectiveness of smartphone-based secondary prevention programs to traditional cardiac rehabilitation in patients with established coronary artery disease | Traditional outpatient-based cardiac rehabilitation | **✓** | **✓** |  |  |  |  |  |
| **Akinosun 2021(12)** | Measure the effectiveness of digital technology (eg, mobile phones, the internet, software applications, wearables, etc) interventions in RCTs | Digital intervention only versus usual care  Digital intervention plus usual care versus usual care | **✓** | **✓** |  |  |  |  |  |

*Composite outcome: Myocardial infarction, stroke, revascularization, hospitalizations, and all-cause mortality

## Supplemental Table 2: Search terms

| **Medline** | | |
| --- | --- | --- |
| **Date of search** | **Volume of papers** | **Search terms** |
| 3-Aug-21 | 298 | 1. (coronary artery disease* or atherosclerosis or acute coronary syndrome or coronary heart disease or heart disease or angina or isch?emic heart disease).mp.  2. (Coronary artery bypass graft* or angioplasty* or revasculari?ation* or percutaneous coronary intervention or heart attack or stent* or myocardial*).mp.  3. Coronary Artery Disease/ or Acute Coronary Syndrome/ or Coronary Disease/ or Heart Diseases/ or Myocardial Ischemia/ or Coronary Artery Bypass/ or Angioplasty/ or Percutaneous Coronary Intervention/ or Myocardial Infarction/ or Stents/ or myocardial revascularization/ 4. (Secondary prevention* or Cardiac rehab* or post discharge support or case management or comprehensive health care or disease management* or disease management program* or rehab*).mp.  5. Cardiac Rehabilitation/ or Secondary Prevention/ or Disease Management/ or Case Management/ or Patient Care Planning/ or Comprehensive Health Care/ or Critical Pathways/  6. (tele* or video* or digital tech* or remote consult* or virtual* or eHealth or mhealth or mobile health or text messag* or online or mobile app*).mp.  7. (smartphone or mobile* or technology or electronic device*).mp.  8. Telemedicine/ or Telerehabilitation/ or Videoconference/ or Telecommunication/ or Remote Consultation/ or wireless technology/ or digital technology/ or Text Messaging/ or cell phone/ or smartphone/ or handheld computer/ or online systems/ or internet based intervention/ or mobile applications/ or Web Browser/ or Web app.mp. 9. (Hospitalization* or readmission* or rehospitalization* or mortality or admission*).mp.  10. Hospitalization/ or patient readmission/ or patient admission/ or mortality/ or survival rate/ 11. 1 or 2 or 3  12. 4 or 5  13. 6 or 7 or 8  14. 9 or 10  15. 11 and 12 and 13 and 14  16. limit 15 to yr="2007 -Current"  17. limit 16 to english language |

| **Embase** | | |
| --- | --- | --- |
| **Date of search** | **Volume of papers** | **Search terms** |
| 3-Aug 21 | 1132 | 1. (coronary artery disease* or atherosclerosis or acute coronary syndrome or coronary heart disease or heart disease or angina or isch?emic heart disease).mp.  2. (Coronary artery bypass graft* or angioplasty* or revasculari?ation* or percutaneous coronary intervention or heart attack or stent* or myocardial*).mp.  3. Coronary Artery Disease/ or Acute Coronary Syndrome/ or Coronary Disease/ or Heart Diseases/ or Myocardial Ischemia/ or Coronary Artery Bypass/ or Angioplasty/ or Percutaneous Coronary Intervention/ or Myocardial Infarction/ or Stents/ or myocardial revascularization/ or coronary artery atherosclerosis/ or atherosclerosis/ or ischemic heart disease/ or heart disease/ or angina pectoris/ or coronary artery bypass graft/ or angioplasty/ or heart muscle revascularization/ or heart infarction/ or stent/ 4. (Secondary prevention* or Cardiac rehab* or post discharge support or case management or comprehensive health care or disease management* or disease management program* or rehab*).mp.  5. Cardiac Rehabilitation/ or Secondary Prevention/ or Disease Management/ or Case Management/ or Patient Care Planning/ or Comprehensive Health Care/ or Critical Pathways/ or heart rehabilitation/ or health program/ 6. (tele* or video* or digital tech* or remote consult* or virtual* or eHealth or mhealth or mobile health or text messag* or online or mobile app*).mp.  7. (smartphone or mobile* or technology or electronic device*).mp.  8. Telemedicine/ or Telerehabilitation/ or Videoconference/ or Telecommunication/ or Remote Consultation/ or wireless technology/ or digital technology/ or Text Messaging/ or cell phone/ or smartphone/ or handheld computer/ or online systems/ or internet based intervention/ or mobile applications/ or telehealth/ or telecardiology/ or teleconsultation/ or telemonitoring/ or teletherapy/ or teleconference/ or teleconsultation/ or virtual rehabilitation system/ or telecommunication/ or telenursing/ or mobile application/ or mobile health application/ or self-care software/ or online system/ or mobile phone/ or personal digital assistant/ or electronic device/  9. (Hospitalization* or readmission* or rehospitalization* or mortality or admission*).mp.  10. hospitalization/ or hospital readmission/ or hospital admission/ or mortality/ or cardiovascular mortality/  11. 1 or 2 or 3  12. 4 or 5  13. 6 or 7 or 8  14. 9 or 10  15. 11 and 12 and 13 and 14  16. limit 15 to yr="2007 -Current"  17. limit 16 to english language |

| **Cochrane central register-controlled trials** | | |
| --- | --- | --- |
| **Date of search** | **Volume of papers** | **Search terms** |
| 3-Aug-21 | 159 | 1. (coronary artery disease* or atherosclerosis or acute coronary syndrome or coronary heart disease or heart disease or angina or isch?emic heart disease).mp.  2. (Coronary artery bypass graft* or angioplasty* or revasculari?ation* or percutaneous coronary intervention or heart attack or stent* or myocardial*).mp.  3. Coronary Artery Disease/ or Acute Coronary Syndrome/ or Coronary Disease/ or Heart Diseases/ or Myocardial Ischemia/ or Coronary Artery Bypass/ or Angioplasty/ or Percutaneous Coronary Intervention/ or Myocardial Infarction/ or Stents/ or myocardial revascularization/  4. (Secondary prevention* or Cardiac rehabilitation or post discharge support or case management or comprehensive health care or disease management* or disease management program* or rehab*).mp.  5. cardiac rehabilitation/ or Secondary Prevention/ or Disease Management/ or Case Management/ or Patient Care Planning/ or Comprehensive Health Care/ or Critical Pathways/ or patient care management/ or patient-centered care/  6. (tele* or videoconferenc* or digital tech* or remote consult* or virtual* or eHealth or mhealth or mobile health or text messag* or online or mobile app*).mp.  7. (smartphone or mobile* or technology or electronic device*).mp.  8. Telemedicine/ or Telerehabilitation.mp. or Videoconferencing/ or Telecommunications/ or remote consultation/ or wireless technology/ or text messaging/ or cellular phone/ or smartphone.mp. or Computers, Handheld/ or online systems/ or internet based intervention.mp. or mobile applications/ or web browser/ or technology/ or telehealth.mp. or telecardiology.mp. or teleconsultation.mp. or telemonitoring.mp. or teletherapy.mp. or teleconference.mp. or telenursing/ or mobile health application.mp. or mobile phone.mp or user-computer interface/ 9. (Hospitalization* or readmission* or rehospitalization* or mortality or admission*).mp.  10. Hospitalization/ or patient readmission/ or patient admission/ or mortality/ or survival rate/ 11. 1 or 2 or 3  12. 4 or 5  13. 6 or 7 or 8  14. 9 or 10  15. 11 and 12 and 13 and 14  16. limit 15 to yr="2007 -Current"  17. limit 16 to english language |

| **CINHAL** | | |
| --- | --- | --- |
| **Date of search** | **Volume of papers** | **Search terms** |
| 3-Aug-21 | 209 | ( (MH "Coronary Arteriosclerosis") OR (MH "Coronary Artery Bypass") OR (MH "Coronary Stenosis") OR (MH "Coronary Disease") OR "coronary artery disease" OR atherosclerosis OR "coronary heart disease" OR (MH "Myocardial Revascularization") OR (MH "Angina Pectoris") OR (MH "Angina, Stable") OR (MH "Angina, Unstable") OR (MH "Coronary Aneurysm") OR (MH "Myocardial Ischemia") OR (MH "Coronary Thrombosis") OR (MH "Acute Coronary Syndrome") OR (MH "Myocardial Infarction") OR (MH "Heart Diseases") OR (MH "Heart Valve Diseases") OR (MH "Angioplasty") OR (MH "Percutaneous Coronary Intervention") OR (MH "Atherectomy, Coronary") OR (MH "Angioplasty, Transluminal, Percutaneous Coronary") OR "heart attack" OR (MH "Stents") OR "myocardial" OR "isch?emic heart disease" )  AND  ( secondary prevention OR (MH "Rehabilitation, Community-Based") OR (MH "Disease Management") OR "disease management" OR (MH "Discharge Planning") OR (MH "Continuity of Patient Care") OR (MH "Case Management") OR (MH "Multidisciplinary Care Team") OR (MH "Preventive Health Care") OR (MH "Rehabilitation, Cardiac") OR "cardiac rehab*" OR (MH "Conditioning, Cardiopulmonary") OR "health program" OR "post discharge support" OR (MH "After Care") OR (MH "Cardiovascular Care") OR "comprehensive healthcare" OR (MH "Health Care Delivery, Integrated") OR "disease management program" OR (MH "Home Rehabilitation") OR "rehab*" OR "patient care planning" OR (MH "Patient Care Plans") OR "heart rehab" )  AND  ( (MH "Telecommunications") OR (MH "Teleconferencing") OR "tele*" OR (MH "Telehealth") OR "telehealth" OR (MH "Intranet") OR (MH "Internet-Based Intervention") OR (MH "Telemedicine") OR "telemedicine" OR (MH "Remote Consultation") OR (MH "Telerehabilitation") OR (MH "Telenursing") OR (MH "Text Messaging") OR (MH "Videoconferencing") OR (MH "Wireless Communications") OR (MH "Internet") OR (MH "Instant Messaging") OR (MH "Online Services") OR (MH "Cellular Phone") OR (MH "Smartphone") OR "smartphone" OR "video*" OR (MH "Digital Technology") OR "digital tech*" OR "remote consult*" OR "virtual*" OR "eHealth" OR "mHealth" OR "mobile health*" OR "remote communication" OR "text messag*" OR "online" OR (MH "Online Systems") OR "online systems" OR (MH "Mobile Applications") OR "mobile app*" OR (MH "Web Browsers") OR (MH "Computers, Hand-Held") OR (MH "Computers, Portable") OR "mobile*" OR "technology" OR "electronic device" OR "telerehab*" OR "videoconferenc*" OR "telecommunication" OR "wireless tech*" OR "cell phone" OR "handheld computer" OR (MH "Rehabilitation Nursing") OR "telecardiology" OR "teleconsultation" OR "telemonitor*" OR "teletherapy" OR "teleconference" OR "virtual rehab*" OR "telenurs*" OR "mobile health app*" OR "personal digital assistant" )  AND  ( hospitilization OR (MH "Readmission") OR "readmission*" OR (MH "Hospitalization") OR (MH "Patient Admission") OR "patient admission" OR "hospitilization*" OR "rehospitilisation*" OR (MH "Mortality") OR "mortality" OR "admission" OR "hospital readmission*" OR "patient readmission" OR "survival rate" )  **Limiters -** Published Date: 20070101-20211231; English Language **Search modes**- Boolean/Phrase |

| **Web of Science** | | |
| --- | --- | --- |
| **Date of search** | **Volume of papers** | **Search terms** |
| 3-Aug-21 | 368 | (((TS=("coronary artery disease" or atherosclerosis or "acute coronary syndrome" or "coronary heart disease" or "heart disease" or angina or "isch?emic heart disease" or "Coronary artery bypass graft*" or angioplasty* or revasculari?ation* or "percutaneous coronary intervention" or "heart attack" or stent* or myocardial* or "Coronary Disease" or "Myocardial Ischemia" or "Myocardial infarction" or "myocardial revascularization" )) AND TS=(( "Secondary prevention" or "Cardiac rehab*" or "health program" or "post discharge support" or "case management" or "comprehensive health care" or "disease management*" or "disease management program*" or rehab* or "Patient Care Planning" or "Critical Pathway*"or "heart rehab*" ) )) AND TS=(( tele* or video* or digital tech* or remote consult* or virtual* or eHealth or mHealth or "mobile health*" or "remote communication*" or "text messag*" or online or "mobile app*" or smartphone or mobile* or "technology" or "electronic device" or telemedicine or Telerehabilitation or videoconferenc* or Telecommunication* or "Remote Consultation*" or "wireless technology" or "digital technology" or "cell phone" or smartphone or "handheld computer" or "online systems" or "internet based intervention" or telehealth or telecardiology or teleconsultation or telemonitoring or teletherapy or teleconference or teleconsultation or "virtual rehabilitation system" or telenurs* or "mobile health application" or "personal digital assistant" or "electronic device" ) )) AND TS=(( Hospitali?ation* or readmission* or rehospitali?ation* or mortality or admission* or "hospital readmission*" or "patient readmission" or "patient admission" or "survival rate" ))  Date: 2007-01-01 to 2021-12-31 Limit to English Language |
| **Scopus** | | |
| **Date of search** | **Volume of papers** | **Search terms** |
| 3-Aug-21 | 85 | ( TITLE-ABS-KEY ( "coronary artery disease"  OR  atherosclerosis  OR  "acute coronary syndrome"  OR  "coronary heart disease"  OR  "heart disease"  OR  angina  OR  "isch?emic heart disease"  OR  "Coronary artery bypass graft*"  OR  angioplasty*  OR  revasculari?ation*  OR  "percutaneous coronary intervention"  OR  "heart attack"  OR  stent*  OR  myocardial*  OR  "Coronary Disease"  OR  "Myocardial Ischemia"  OR  "Myocardial infarction"  OR  "myocardial revascularization" )   AND   TITLE-ABS-KEY ( "Secondary prevention"  OR  "Cardiac rehab*"  OR  "health program*"  OR  "post discharge support"  OR  "case management"  OR  "comprehensive health care"  OR  "disease management*"  OR  "disease management program*"  OR  rehab*  OR  "Patient Care Planning"  OR  "Critical Pathway*"  OR  "heart rehab*" )  AND   TITLE-ABS KEY ( tele*  OR  video*  OR  digital  AND tech*  OR  remote  AND consult*  OR  virtual*  OR  ehealth  OR  mhealth  OR  "mobile health*"  OR  "remote communication*"  OR  "text messag*"  OR  online  OR  "mobile app*"  OR  smartphone  OR  mobile*  OR  "technology"  OR  "electronic device"  OR  telemedicine  OR  telerehabilitation  OR  videoconferenc*  OR  telecommunication*  OR  "Remote Consultation*"  OR  "wireless technology"  OR  "digital technology"  OR  "cell phone"  OR  "cellular phone"  OR  smartphone  OR  "handheld computer"  OR  "online system*"  OR  "internet based intervention"  OR  telehealth  OR  telecardiology  OR  teleconsultation  OR  telemonitoring  OR  teletherapy  OR  teleconference  OR  teleconsultation  OR  "virtual rehabilitation system"  OR  telenurs*  OR  "mobile health application"  OR  "personal digital assistant"  OR  "electronic device" )   AND   TITLE-ABS-KEY ( hospitali?ation*  OR  readmission*  OR  rehospitali?ation*  OR  mortality  OR  admission*  OR  "hospital readmission*"  OR  "patient readmission"  OR  "patient admission"  OR  "survival rate" ) )   AND   PUBYEAR  >  2006  AND  ( LIMIT-TO ( LANGUAGE ,  "English" ) ) |
| **Grey literature** | | |
| **Date of search** | **Volume of papers** | **Search terms** |
| 9/21/2021 - end of October 2021 | **1088** papers (excluding duplicates)  **466** papers added after removing duplicates  plus an additional 21 papers added from additional grey literature search | Screened the reference list of 43 systematic reviews that were picked up within our search. Went through each reference lists and downloaded the relevant papers and added them back into COVIDENCE. This was a broad, robust and far reaching approach, providing confidence that all relevant papers have been captured.   Additionally, we searched through 48 relevant protocol papers to see if the results papers have been published and added any results papers back into Covidence for screening.   A total of 1088 additional papers were added via this method with 466 remaining after removing duplicates. We then added an additional 21 papers to screening after more grey literature searching. This was undertaken due to finding conference abstracts so we undertook additional searchers per abstract to find the published papers. |

## Supplemental Figure 1: Funnel plots and Eggers test to assess publication bias

| 1. **Funnel plot: All-cause readmissions**   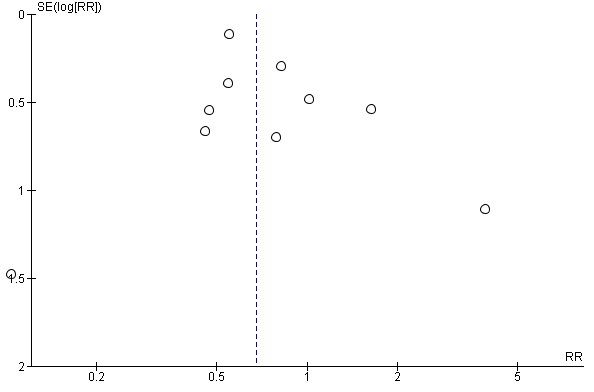 | 1. **Funnel plot: MACE**   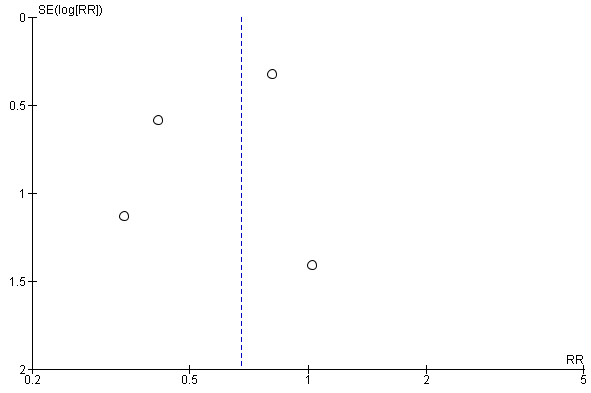 |
| --- | --- |
| 1. **Funnel plot: cardiac related readmissions**   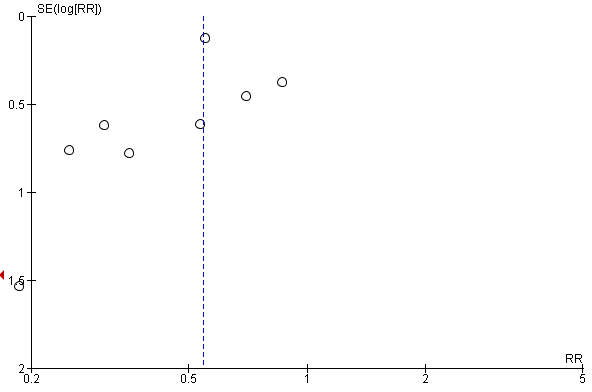 | 1. **Funnel plot: All-cause mortality**   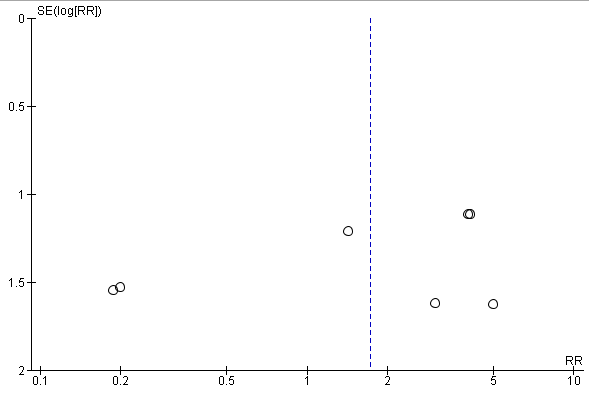 |
| 1. **Funnel plot: Ed visits**   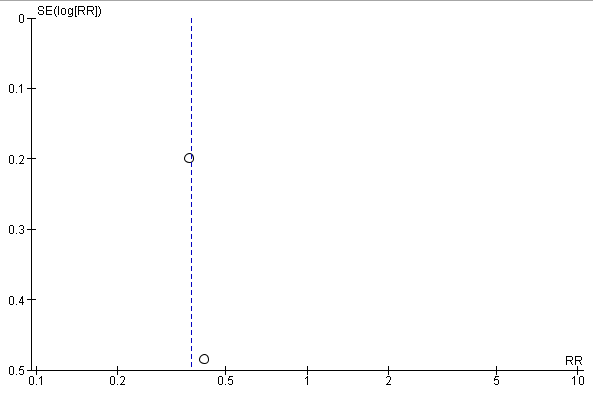 |  |

There was no evidence of funnel plot asymmetry or significant Egger tests and thus no evidence of publication bias.

| **Egger Test beta (p value)** | |
| --- | --- |
| All_Cause_Rehosp | 0.59 (0.2161) |
| All_Cause_mortality | -1.96 (0.2903) |
| Cardiac_related_Rehosp | Convergence not achieved during tau2 estimation |
| MACE | -0.56 (0.5753) |
| ED | 0.45 (1.0000) |

## Supplemental Table 3: Pooled intervention characteristics

|  | **Intervention characteristic** | **Number of studies (%)** |
| --- | --- | --- |
| **Setting**  **(in addition to home-based)** | Centre-based | 10 (56%) |
|  | Hybrid | 8 (44%) |
| **Mode of delivery**  **(in conjunction with remote delivery)** | Face to face | 9 (50%) |
|  | 1:1 care# | 10 (56%) |
|  | Combination of 1:1 and group-based treatment | 8 (44%) |
|  | Group based | 0 (0%) |
|  | Personalized treatment** | 10 (56%) |
|  | Semi-personalized treatment †† | 8 (44%) |
| **Type of provider delivering care** | Nurse alone | 3 (17%) |
|  | Exercise physiologist alone | 2 (11%) |
|  | Multidisciplinary team | 3 (17%) |
|  | No provider/health care professional (such as digital automated messages only) | 3 (17%) |
|  | Unknown (not specified) | 7 (39%) |
| **Disease management program components** | Multicomponent DMP (defined as having 5 or more DMP components) | 15 (83%) |
|  | Simple DMP (defined as having 4 or less DMP components) | 3 (16%) |

**Notes:**

# 1:1 setting= non group setting and Combination setting= part of intervention is 1:1, and part was traditional group-based setting

**Personalized = parts of the intervention/ care delivery were tailored and personalized according to the specific needs or specific conditions of the patient.

††Semi-personalized = the care delivery was partly tailored to the individual such as specific messages based on risk factors that the patient has (but the same message went to all patients who have that risk factor, eg smokers see all the same smoking content).

## Supplemental Table 4: Baseline characteristics of included studies

| **First author** | **Year** | **Sample size**  **(n)** | | **Age**  **(mean, SD) years** | | **Male**  **(%)** | | **Current smoker**  **(%)** | | **Diabetes**  **(%)** | | **Hypertension (%)** | | **BMI**  **(mean, SD)** | | **Total cholesterol**  **(mean, SD) mmol/L** | | **LDL cholesterol**  **(mean, SD) mmol/L** | | **HDL cholesterol**  **(mean, SD) mmol/L** | | **SBP**  **(mean, SD)**  **mmHg** | | **DBP**  **(mean, SD)**  **mmHg** | |
| --- | --- | --- | --- | --- | --- | --- | --- | --- | --- | --- | --- | --- | --- | --- | --- | --- | --- | --- | --- | --- | --- | --- | --- | --- | --- |
|  |  | **Intervention** | **Control** | **Intervention** | **Control** | **Intervention** | **Control** | **Intervention** | **Control** | **Intervention** | **Control** | **Intervention** | **Control** | **Intervention** | **Control** | **Intervention** | **Control** | **Intervention** | **Control** | **Intervention** | **Control** | **Intervention** | **Control** | **Intervention** | **Control** |
| **Bae** | 2021 | 440 | 439 | 60.1 ± 10.6 | 60.7 ± 10.4 | 84 | 83 | 43 | 42 | 29 | 29 | 48 | 48 | 25 ± 3.4 | 24.9 ± 3.1 |  |  | 2.9 ± 1.1 | 2.8 ± 1.0 |  |  | 124 ± 19 | 126 ± 20 | 74 ± 12 | 75 ± 13 |
| **Chow** | 2015 | 352 | 358 | 57.9 ± 9.1 | 57.3 ± 9.3 | 82 | 82 | 52 | 54 | 32 | 33 | 63 | 61 | 29.8 ± 6 | 29.6 ± 5.9 | 4.6 ± 1.2 | 4.6 ± 1.1 | 2.7 ± 1.0 | 2.6 ± 0.9 | 1.0 ± 0.2 | 1.1 ± 0.3 | 129 ± 12 | 129 ± 12 | 83 ± 8 | 83 ± 7 |
| **Frederix** | 2015 | 40 | 40 | 58 | 63 ± 10 | 81 | 85 | 9 | 15 | 25 | 24 | 63 | 38 | 29.1 ± 4.9 | 26.8 ± 3.6 | 3.3 ± 0.8 | 3.6 ± 0.8 | 1.7 ± 0.6 | 1.8 ± 0.6 | 1.1 ± 0.2 | 1.2 ± 0.4 |  |  |  |  |
| **Frederix** | 2017 | 62 | 64 | 61 ± 9 | 61 ± 8 | 84 | 80 | 45 | 39 |  |  | 56 | 63 |  |  |  |  |  |  |  |  |  |  |  |  |
| **Khonsari** | 2015 | 31 | 31 | 56 ± 11.3 | 59 ± 13.9 | 87 | 84 |  |  |  |  |  |  |  |  |  |  |  |  |  |  |  |  |  |  |
| **Khonsari** | 2020 | 39 | 39 | 60.4 ± 1.6 | 63.3 ± 1.3 | 72 | 72 |  |  |  |  |  |  |  |  |  |  |  |  |  |  |  |  |  |  |
| **Kraal** | 2017 | 45 | 45 | 60.5 ± 8.8 | 57.7 ± 8.7 | 89 | 89 |  |  |  |  |  |  | 27.8 ± 4.8 | 28.2 ± 3.9 |  |  |  |  |  |  |  |  |  |  |
| **Maddison** | 2018 | 82 | 80 | 61 ± 13.2 | 61.5 ± 12.2 | 84 | 88 | 0 | 1 | 18 | 18 | 65 | 61 | 29.09 ± 4.6 | 27.94 ± 3.5 | 3.39 ± 0.8 | 3.5 ± 0.8 | 1.8 ± 0.7 | 1.7 ± 0.6 | 1.1 ± 0.4 | 1.1 ± 0.4 | 139 ± 17 | 134 ± 17 | 81 ± 11 | 80 ± 10 |
| **Mcelroy** | 2016 | 27 | 416 | 62.9 ± 9.8 | 65.9 ± 14.1 | 85 | 66 | 33 | 33 | 26 | 24 | 70 | 55 | 31.2 ± 14.0 | 28.6 ± 19.8 |  |  |  |  |  |  |  |  |  |  |
| **Pakrad** | 2021 | 44 | 44 | 62.6 ± 8.1 | 62.9 ± 9.8 | 82 | 86 | 11 | 14 | 45 | 36 | 66 | 70 | 26.3 ± 3.7 | 26.78 ± 4.3 | 4.3 ± 1.1 | 3.9 ± 1.2 | 2.3 ± 0.9 | 2.3 ± 0.9 | 0.9 ± 0.2 | 0.9 ± 0.2 |  |  |  |  |
| **Pfaeffli** | 2015 | 61 | 62 | 59 ±10.5 | 59.9 ± 11.8 | 79 | 84 | 20 | 18 | 23 | 11 |  |  | 31 ± 6.4 | 28.0 ± 4.2 | 4.6 ± 1.2 | 4.3 ± 1.2 | 2.7 ± 1.3 | 2.4 ± 1 | 1.1 ± 0.3 | 1.1 ± 0.3 | 131 ± 17 | 129 ± 26 | 78 ± 11 | 75 ± 11 |
| **Reid** | 2012 | 115 | 108 | 56.7 ± 9 | 56 ± 9 | 83 | 86 | 11 | 18 | 17 | 20 |  |  | 28.9 ± 4.4 | 29.6 ± 5.2 |  |  |  |  |  |  |  |  |  |  |
| **Riegel** | 2020 | 62 | 68 | 59.5 ± 11.3 | 57.3 ± 10 | 60 | 63 |  |  |  |  |  |  |  |  |  |  |  |  |  |  |  |  |  |  |
| **Snoek** | 2019 | 61 | 61 | 60 ± 8.4 | 59 ± 10.7 | 82 | 82 | 7 | 11 | 13 | 13 | 39 | 52 | 28.1 ± 3.6 | 28.4 ± 3.7 | 4.1 ± 0.9 | 4.3 ± 1.2 | 2.2 ± 0.6 | 2.3 ± 1 |  |  | 128 ± 16 | 128 ± 15 | 79 ± 9 | 79 ± 9 |
| **Widmer** | 2017 | 37 | 34 | 62.5 ± 10.7 | 63.6 ± 10.9 | 78 | 85 | 3 | 15 | 30 | 12 | 76 | 62 | 31.4 ± 5 | 30.5 ± 6 | 4.6 ± 1.2 | 4.7 ± 1.0 | 2.5 ± 1.0 | 2.7 ± 0.7 | 1.2 ± 0.3 | 1.1 ± 0.3 | 119 ± 16 | 116 ± 13 | 67 ± 11 | 66 ± 9 |
| **Wolf** | 2016 | 37 | 105 | 59.8 ± 10.1 | 61.3 ± 8.9 | 81 | 70 |  |  |  |  |  |  |  |  |  |  |  |  |  |  |  |  |  |  |
| **Woodend** | 2008 | 62 | 66 | 66 ± 12 | 65 ± 10 | 77 | 79 |  |  |  |  |  |  |  |  |  |  |  |  |  |  |  |  |  |  |
| **Yudi** | 2021 | 83 | 85 | 56.8 ± 9.9 | 56.2 ± 10.2 | 86 | 84 | 30 | 28 | 22 | 15 | 47 | 44 | 29.7 ± 6.1 | 29.9 ± 4.7 | 4.8 ± 1.3 | 4.9 ± 1.3 | 2.9 ± 1.1 | 3 ± 1.2 | 1.1 ± 0.3 | 1.1 ± 0.2 | 123.0 ± 14.9 | 121 ± 14 | 73 ± 10 | 72 ± 11 |
| **Pooled baseline characteristics, weighted average** | | **1,680** | **2,145** | **60.3** | **62.6** | **82** | **80** | **24** | **19** | **26** | **24** | **56** | **55** | **28.2** | **27.7** | **4.0** | **4.1** | **2.2** | **2.7** | **1.0** | **1.1** | **127** | **125** | **77** | **76** |

SD= Standard deviation

BMI = body mass index

LDL = Low density lipoprotein cholesterol

HDL = high density lipoprotein cholesterol

SDP = systolic blood pressure

DPB =diastolic blood pressure

## Supplemental Table 5: Meta-regression variables and results

|  | **Statistically significant covariates from meta regression** | **P-value of coefficient** |
| --- | --- | --- |
| All-Cause readmission |  |  |
|  | Intev_age | 0.116 |
|  | Intev_Control_current_smkr_%_log_odds_diff | 0.203 |
|  | Intev_Control_num_male_%_logg_odds_diff | 0.405 |
|  | Intev_Control_diabetes_%_log_odds_diff | 0.830 |
|  | Average_cohort_year1 (intervention) | 0.133 |
|  | Average_cohort_year2(control) | 0.143 |
| All-Cause mortality |  |  |
|  | Intev_age | 0.231 |
|  | Intev_Control_current_smkr_%_log_odds_diff | 0.075 |
|  | Intev_Control_num_male_%_logg_odds_diff | 0.602 |
|  | Intev_Control_diabetes_%_log_odds_diff | 0.212 |
|  | Average_cohort_year1 (intervention) | 0.812 |
|  | Average_cohort_year2(control) | 0.688 |
| Cardiac-related readmission |  |  |
|  | Intev_age | 0.593 |
|  | Intev_Control_current_smkr_%_log_odds_diff | 0.470 |
|  | Intev_Control_num_male_%_logg_odds_diff | 0.382 |
|  | Intev_Control_diabetes_%_log_odds_diff | 0.555 |
|  | intev_control_hypertension_%_log_odds_diff | 0.527 |
|  | interv_control_familyhx_cvd_%_log_odds_diff | 0.460 |
|  | interv_control_ace_inhibitor_%_log_odds_diff | 0.416 |
|  | interv_control_blocker_%_log_odds_diff | 0.914 |
|  | interv_control_statin_%_log_odds_diff | 0.852 |
|  | Average_cohort_year1 (intervention) | 0.226 |
|  | Average_cohort_year2(control) | 0.156 |
| MACE |  |  |
|  | Intev_age | 0.455 |
|  | Intev_Control_current_smkr_%_log_odds_diff | 0.992 |
|  | Intev_Control_num_male_%_logg_odds_diff | 0.342 |
|  | Intev_Control_diabetes_%_log_odds_diff | 0.390 |
|  | intev_control_total_chol_mean_mmoll_diff | 0.263 |
|  | intev_control_ldl_mean_mmoll_diff | 0.238 |
|  | intev_control_sbp_mmhg_mean_diff | 0.763 |
|  | intev_control_dbp_mmhg_mean_diff | 0.763 |
|  | intev_control_hypertension_%_log_odds_diff | - |
|  | interv_control_pci_%_log_odds_diff | 0.850 |
|  | Average_cohort_year1 (intervention) | 0.408 |
|  | Average_cohort_year2 (control) | 0.272 |
| ED |  |  |
|  | Intervention age | * |
|  | Intev_Control_current_smkr_%_log_odds_diff | * |
|  | Intev_Control_num_male_%_logg_odds_diff | * |
|  | Intev_Control_diabetes_%_log_odds_diff | * |
|  | Average_cohort_year1 (intervention) | * |
|  | Average_cohort_year2 (control) | * |

* Convergence not achieved during tau2 estimation

## Supplemental Table 6: Sub-group analysis per study outcome (p-values)

|  | **All-cause readmission** | **All-Cause mortality** | **Cardiac related readmission** | **MACE** | **ED visits** |
| --- | --- | --- | --- | --- | --- |
| Duration of intervention | 0.12 | 0.541 | 0.962 | 0.763 | 0.808 |
| Length of follow-up | 0.32 | 0.58 | 0.54 | 0.77 | - |
| Year of publication (pre 2017 or post 2017) | 0.964 | 0.408 | 0.479 | 0.353 | 0.808 |
| Age less than 60 years  mHealth enabled DMPs  standard DMP | 0.116  0.15 | 0.23  0.387 | 0.593  0.535 | 0.455  0.536 | - |
| BMI  mHealth enabled DMPs  standard DMP | 0.67  0.728 | 0.616 | 0.308  0.757 | -  0.786 | - |
| Exercise prescription | 0.4 | 0.13 | 0.28 | 0.54 | - |
| Health professional coaching/consultations | 0.58 | 0.69 | 0.28 | 0.54 | 0.81 |
| Medication management | 0.67 | 0.4 | 0.79 | 0.77 | 0.81 |
| Monitoring of health metrics | - | 0.69 | 0.277 | 0.536 | - |
| Psychosocial support | 0.189 | 0.097 | 0.139 | 0.544 | - |
| Goal setting | 0.149 | 0.894 | 0.299 | 0.536 | - |
| Multidisciplinary team | 0.106 | 0.532 | 0.606 | 0.238 | - |
| Mobile applications | 0.704 | 0.793 | 0.973 | - | 0.808 |
| SMS text messages | 0.09 | 0.795 | 0.589 | 0.766 | 0.808 |
| Websites | 0.091 | 0.224 | 0.882 | - | - |
| Web application / provider dashboard | 0.052 | 0.152 | 0.447 | 0.763 | 0.808 |
| Teleconference | 0.921 | 0.681 | 0.589 | 0.311 | 0.808 |
| Mobile iPads/electronic tablets | 0.108 | 0.483 | 0.493 | 0.353 | - |
| Wearables/monitoring devices | 0.545 | 0.674 | 0.696 | 0.766 | - |
| Computers (desktop or laptop) | 0.317 | 0.3 | 0.201 | 0.238 | - |

## Supplemental Figure 2: Risk of Bias Assessment

***a) Randomized controlled trials***

**
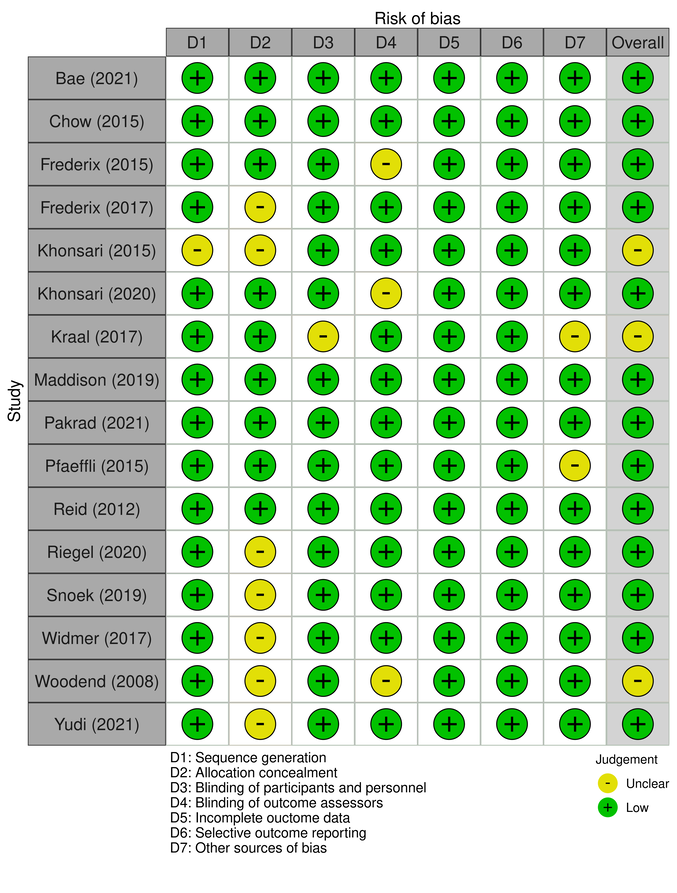
**

***b) Summary plot (randomized controlled trials)***

***
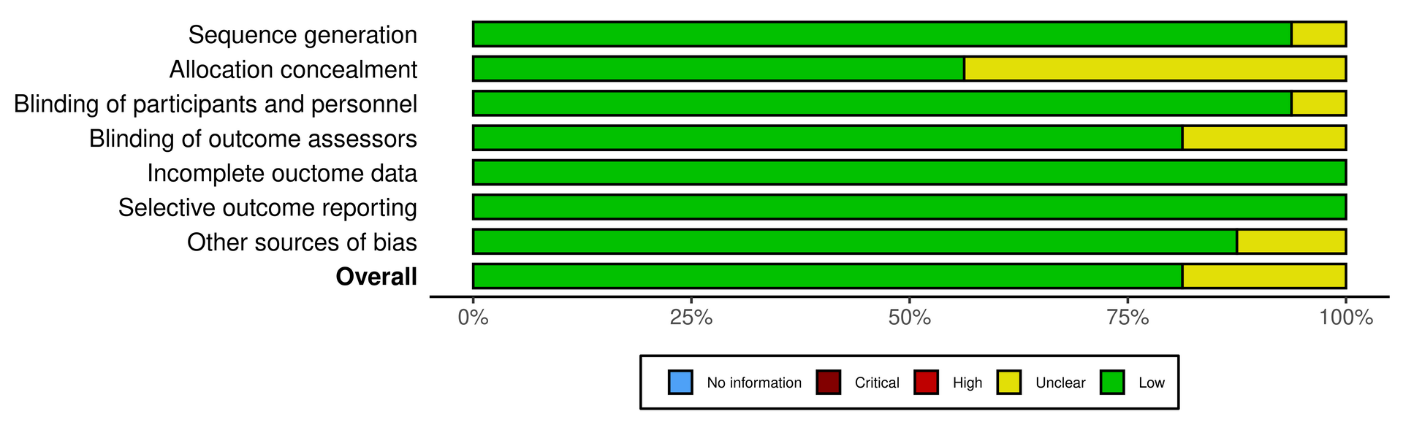
***

***c) Observational studies***

***
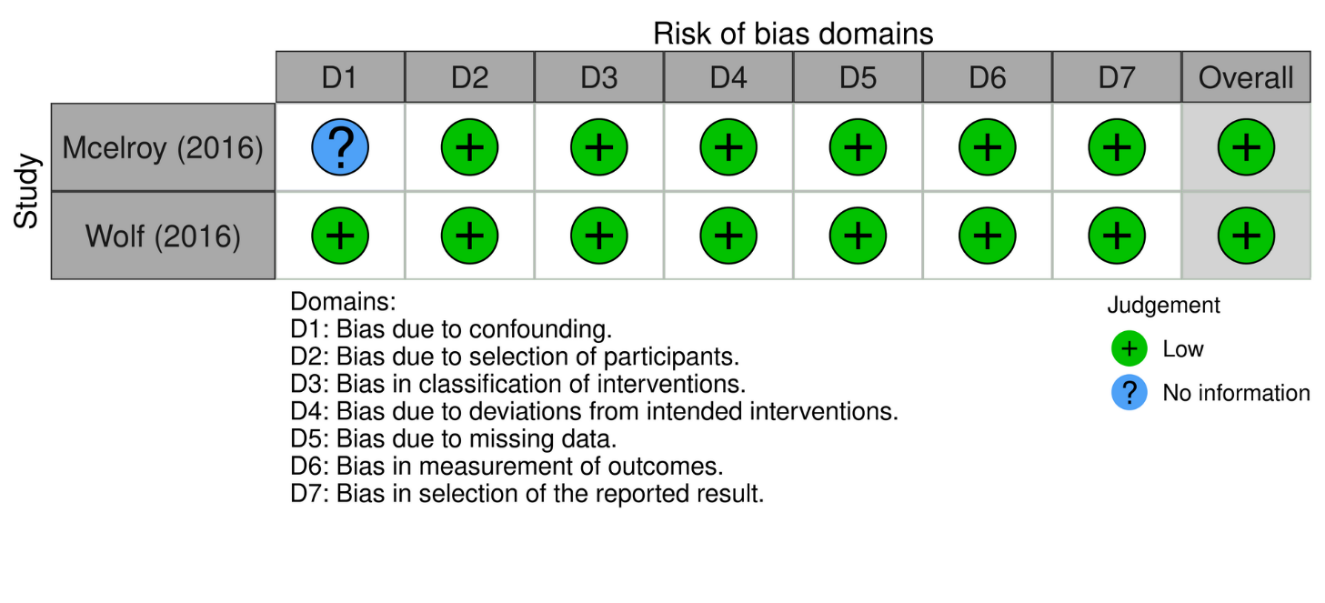
***

***d) Summary plot (observational studies)***

***
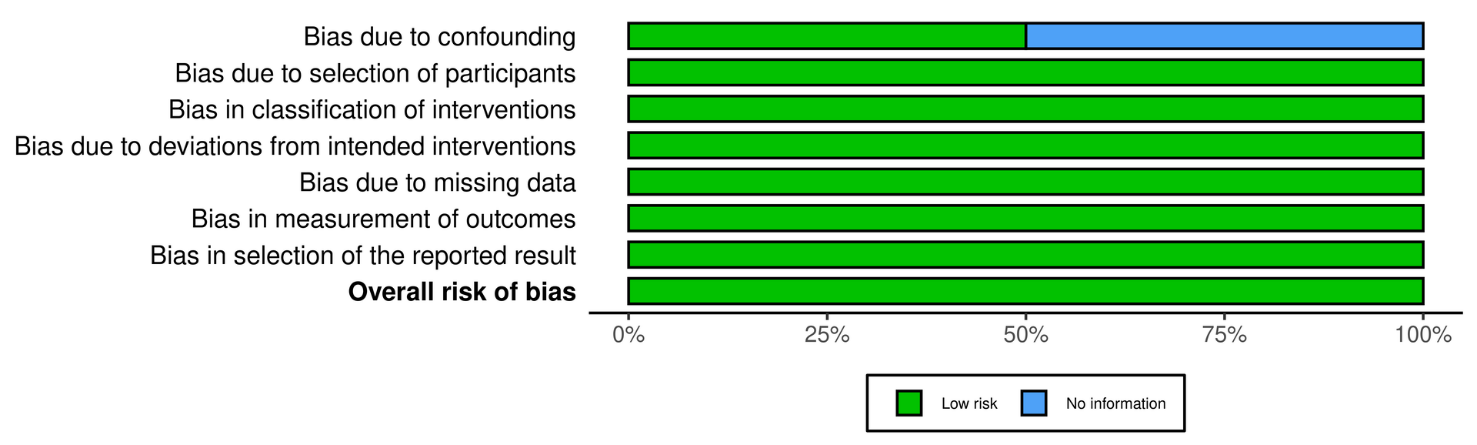
***

***a) Randomized controlled trials***

Risk of bias plots were generated using robis (13) and the risk of bias was assessed using The Cochrane Collaboration’s tool (14) for randomized controlled trials (n=16). The risk of bias across domains for each study was judged to be low or unclear.

***b) Summary plot (randomized controlled trials)***

The overall risk of bias across all domains of The Cochrane Collaboration’s tool (14) for randomized controlled trials was judged to be low or unclear.

***c) Observational studies***

Risk of bias plots were generated using robis (13) and the ROBINS-I assessment tool (15) was used to assess the risk of bias for observational studies (n=2). The risk of bias across domains for each study was judged to be low or unclear.

***d) Summary plot (observational studies)***

The overall risk of bias across all domains of the ROBINS-I assessment tool (15) was judged to be low or unclear.

## Supplemental Table 7: Grade Profile Quality Assessment

| **Certainty assessment** | | | | | | | **Summary of findings** | | | | |
| --- | --- | --- | --- | --- | --- | --- | --- | --- | --- | --- | --- |
| **Participants (studies) Follow-up** | **Risk of bias** | **Inconsistency** | **Indirectness** | **Imprecision** | **Publication bias** | **Overall certainty of evidence** | **Study event rates (%)** | | **Relative effect (95% CI)** | **Anticipated absolute effects** | |
|  |  |  |  |  |  |  | **With Traditional DMPs alone** | **With mHealth DMPs** |  | **Risk with Traditional DMPs alone** | **Risk difference with mHealth DMPs** |
| **All-cause readmissions** | | | | | | | | | | | |
| 1514 (10 RCTs)^a^ | not serious | not serious^b^ | serious^c^ | not serious^d^ | none | ⨁⨁⨁◯ Moderate | 180/989 (18.2%) | 84/525 (16.0%) | **RR 0.68** (0.50 to 0.91) | 18 per 100 | **6 fewer per 100** (from 9 fewer to 2 fewer) |
| **Cardiac-related readmissions** | | | | | | | | | | | |
| 1009 (9 RCTs) | not serious | not serious^e^ | not serious^f^ | not serious^d^ | none | ⨁⨁⨁⨁ High | 119/501 (23.8%) | 61/508 (12.0%) | **RR 0.55** (0.44 to 0.68) | 24 per 100 | **11 fewer per 100** (from 13 fewer to 8 fewer) |
| **ED visits** | | | | | | | | | | | |
| 199 (2 RCTs) | not serious | not serious^e^ | not serious^g^ | serious^h^ | strong association^i^ | ⨁⨁⨁⨁ High | 66/100 (66.0%) | 24/99 (24.2%) | **RR 0.37** (0.26 to 0.54) | 66 per 100 | **42 fewer per 100** (from 49 fewer to 30 fewer) |
| **MACE** | | | | | | | | | | | |
| 588 (4 RCTs) | not serious | not serious^j^ | serious^k^ | serious^l^ | none | ⨁⨁◯◯ Low | 29/292 (9.9%) | 19/296 (6.4%) | **RR 0.68** (0.40 to 1.15) | 10 per 100 | **3 fewer per 100** (from 6 fewer to 1 more) |
| **All-cause mortality** | | | | | | | | | | | |
| 2711 (8 RCTs)^m^ | not serious | serious^n^ | serious^k,o^ | serious^p^ | none | ⨁◯◯◯ Very low | 9/1582 (0.6%) | 10/1129 (0.9%) | **RR 1.72** (0.64 to 4.64) | 1 per 100 | **0 fewer per 100** (from 0 fewer to 2 more) |

**CI:** confidence interval; **RR:** risk ratio

#### Explanations

a. 8 out of the 10 studies were RCTs and 2 were non RCTs. 80% or more are RCTs therefore quality of study design is not downgraded.

b. i^2=23%; CIs overlap and overall finding is significant treatment effect. Yet there is some inconsistency in the CIs across the treatment effect.

c. Indirect interventions comparisons, most of the interventions were similar and comparable but two studies had varying interventions compared to the pooled studies.

d. Sufficient sample size (number of events exceeds 100) and significant treatment effect with small CIs.

e. i^2=0%; CIs overlap and are all skewed in favor of intervention. Overall finding is significant treatment effect in favor of mHealth DMP

f. Population, interventions, comparison and outcomes were similar between most of the studies. Only one study out of the nine included studies had a less comprehensive intervention (Khonsari 2015), which focused on medication reminder sms. All other interventions included Health professional consultations; education; exercise prescription; metrics monitoring; self management; behavior change; goal setting and all had similar components, durations and intensities.

g. Population, intervention, comparison and outcomes were similair across the two included studies. Interventions varied slightly, both studies had same duration and included health professional consultations; education; metrics monitoring; self management but the digital components differed. One study included mobile application; email; web based portal; telemonitoring; online messaging; and the other included telemonitoring and videoconferencing. Therefore there is direct comparability.

h. Small sample size, number of events is less than 100 (the pooled results equate to 90 events). However there is a significant treatment effect with small CIs.

i. The effect was large (RR=0.37) and is <0.5 based on consistent evidence from at least 2 studies, with no plausible confounders. Therefore large effect is chosen.

j. i^2=0%; CIs overlap and are all skewed in favor of intervention. However, overall finding is not significant

k. indirect interventions comparisons

l. Wide CI; insufficient sample size n=48 events in total

m. Two out of the eight studies are observational studies.

n. Variation in association estimates

o. Many studies did not report on mortality as a primary or secondary outcome and many of the interventions were designed to address clinical and behavioural health outcomes and not reduce mortality. Despite this, we extracted all available (post baseline assessment) mortality data. This may have conservatively underestimated the treatment effect on mortality.

p. Insufficient sample size n=19 events in total; wide CIs and uncertainty about magnitude of effect

## Supplemental Table 8: DMP alone (control group) characteristics per study

| **Study** | **Type of comparison** | **Setting** | **Mode of delivery** | **Human consultations** | **Education** | **Medication management** | **Exercise prescription** | **Monitoring of health metrics** | **Self-management** | **Psychosocial** | **Behaviour change** | **Goal setting** | **Multidisciplinary team** |
| --- | --- | --- | --- | --- | --- | --- | --- | --- | --- | --- | --- | --- | --- |
| **Bae (2021)** | DMP standard care - med management & regular follow-up | Outpatient care | F2F | ✔ | ✔ | ✔ | ✖ | ✖ | ✔ | ✖ | ✖ | ✖ | ✖ |
| **Chow (2015)** | Traditional phase 2 inpatient CR | Centre based | F2F | ✔ | ✔ | ✖ | ✔ | ✖ | ✔ | ✔ | - | ✔ | ✔ |
| **Frederix (2015)** | Traditional phase 2 CR followed by no intervention for phase 3 CR | Centre based | F2F & hybrid | ✖ | ✔ | ✖ | ✔ | ✔ | ✔ | ✔ | ✖ | ✖ | ✖ |
| **Frederix (2017)** | Traditional phase 2 CR followed by no intervention | Centre based | F2F | ✔ | ✔ | - | ✔ | ✖ | ✔ | ✖ | ✖ | ✖ | ✔ |
| **Khonsari (2015)** | Traditional CR+ outpatient cardiology appointment | Centre based | F2F | ✔ | ✔ | ✔ | ✖ | ✖ | ✖ | ✖ | ✖ | ✖ | ✖ |
| **Khonsari (2020)** | Traditional phase 2 exercise CR | Centre based | F2F | ✔ | ✔ | ✖ | ✔ | ✖ | ✔ | ✔ | ✖ | ✖ | ✖ |
| **Kraal (2017)** | Traditional Centre based CR for 12 weeks | Centre based | F2F | ✔ | ✔ | ✖ | ✔ | ✔ | ✔ | ✖ | ✖ | ✖ | ✔ |
| **Maddison (2019)** | Traditional phase 2 exercise CR delivered for 12 weeks | Centre based | F2F | ✔ | ✔ | ✔ | ✔ | ✔ | ✔ | ✔ | ✔ | ✔ | ✔ |
| **Mcelroy (2016)** | Traditional/formal risk reduction DMP | Home | Remote | ✔ | ✔ | ✔ | ✖ | ✖ | ✔ | ✖ | ✖ | ✖ | ✖ |
| **Pakrad (2021)** | Traditional centred based 12 week CR | Centre based | F2F | ✔ | ✔ | ✖ | ✔ | ✖ | ✔ | ✖ | ✖ | ✖ | ✖ |
| **Pfaeffli 2015)** | Traditional 6 week F2F CR | Centre based | F2F | ✔ | ✔ | ✔ | ✔ | ✖ | ✖ | ✔ | ✖ | ✖ | ✖ |
| **Reid (2012)** | DMP with physical activity guidance from cardiologist + education booklet | Outpatient care | F2F | ✔ | ✔ | ✖ | ✔ | ✖ | ✔ | ✖ | ✖ | ✖ | ✖ |
| **Riegel (2020)** | Medication management intervention with no financial incentives or mobile app | Home | Remote | ✖ | ✖ | ✔ | ✖ | ✔ | ✔ | ✖ | ✖ | ✖ | ✖ |
| **Snoek (2019)** | Traditional phase 2 CR | Outpatient care | F2F | ✔ | ✖ | ✖ | ✖ | ✖ | ✖ | ✖ | ✖ | ✖ | ✖ |
| **Widmer (2017)** | Traditional F2F CR | Centre based | F2F | ✔ | ✔ | ✔ | ✔ | ✖ | ✖ | ✖ | ✖ | ✖ | ✖ |
| **Wolf (2016)** | Traditional DMP | Outpatient care | F2F | ✔ | ✔ | ✔ | ✖ | ✖ | ✖ | ✖ | ✖ | ✖ | ✔ |
| **Woodend (2008)** | Home Care and / or Outpatient care by community physician or cardiologist | Home & Outpatient care | F2F | ✔ | ✖ | ✖ | ✖ | ✖ | ✖ | ✖ | ✖ | ✖ | ✖ |
| **Yudi (2021)** | Traditional secondary prevention rehab | Home & Outpatient care | F2F | ✔ | ✔ | ✖ | ✖ | ✖ | ✔ | ✖ | ✖ | ✖ | ✔ |

## Supplemental Table 9: Summary of DMP alone (control group) characteristics

|  | **Intervention characteristic** | **Total** |
| --- | --- | --- |
| **Type of DMP** | Traditional cardiac rehab | 10 (56%) |
|  | Traditional exercise based cardiac rehab | 2 (11%) |
|  | Other type of DMP | 6 (33%) |
| **Setting** | Home | 2 (11%) |
|  | Centre-based | 10 (56%) |
|  | Outpatient care | 4 (22%) |
|  | Hybrid: Home & Outpatient care | 2 (11%) |
| **Mode of delivery** | Remotely delivered | 2 (11%) |
|  | Face to face | 15 (83%) |
|  | Hybrid (F2F & remote) | 1 (6%) |
| **Disease management program components** | Multicomponent DMP (defined as having 5 or more DMP components) | 8 (44%) |
|  | Simple DMP (defined as having 4 or less DMP components) | 10 (56%) |

**References**

1. Neubeck L, Redfern JU, Fernandez R, Briffa T, Bauman A, Freedman SB. Telehealth interventions for the secondary prevention of coronary heart disease: a systematic review. European Journal of Cardiovascular Prevention & Rehabilitation 2009;16:281-289.

2. Widmer RJ, Collins NM, Collins CS, West CP, Lerman LO, Lerman A. Digital Health Interventions for the Prevention of Cardiovascular Disease: A Systematic Review and Meta-analysis. Mayo Clinic Proceedings 2015;90:469-480.

3. Clark RA, Conway A, Poulsen V, Keech W, Tirimacco R, Tideman P. Alternative models of cardiac rehabilitation: A systematic review. European Journal of Preventive Cardiology 2015;22:35-74.

4. Huang K, Liu W, He D et al. Telehealth interventions versus center-based cardiac rehabilitation of coronary artery disease: A systematic review and meta-analysis. European Journal of Preventive Cardiology 2015;22:959-971.

5. Gandhi S, Chen S, Hong L et al. Effect of Mobile Health Interventions on the Secondary Prevention of Cardiovascular Disease: Systematic Review and Meta-analysis. Can J Cardiol 2017;33:219-231.

6. Hamilton SJ, Mills B, Birch EM, Thompson SC. Smartphones in the secondary prevention of cardiovascular disease: a systematic review. BMC Cardiovascular Disorders 2018;18.

7. Jin K, Khonsari S, Gallagher R et al. Telehealth interventions for the secondary prevention of coronary heart disease: A systematic review and meta-analysis. European Journal of Cardiovascular Nursing 2019;18:260-271.

8. Su JJ, Yu DSF, Paguio JT. Effect of eHealth cardiac rehabilitation on health outcomes of coronary heart disease patients: A systematic review and meta-analysis. J Adv Nurs 2020;76:754-772.

9. Wongvibulsin S, Habeos EE, Huynh PP et al. Digital Health Interventions for Cardiac Rehabilitation: Systematic Literature Review. Journal of Medical Internet Research 2021;23:e18773.

10. Subedi N, Rawstorn JC, Gao L, Koorts H, Maddison R. Implementation of Telerehabilitation Interventions for the Self-Management of Cardiovascular Disease: Systematic Review. JMIR mHealth and uHealth 2020;8:e17957.

11. Murphy AC, Meehan G, Koshy AN, Kunniardy P, Farouque O, Yudi MB. Efficacy of Smartphone-Based Secondary Preventive Strategies in Coronary Artery Disease. Clinical Medicine Insights: Cardiology 2020;14:117954682092740.

12. Akinosun AS, Polson R, Diaz - Skeete Y et al. Digital Technology Interventions for Risk Factor Modification in Patients With Cardiovascular Disease: Systematic Review and Meta-analysis. JMIR mHealth and uHealth 2021;9:e21061.

13. McGuinness LA, Higgins JPT. Risk-of-bias VISualization (robvis): An R package and Shiny web app for visualizing risk-of-bias assessments. Research Synthesis Methods 2020;n/a.

14. Higgins JPT, Altman DG, Gotzsche PC et al. The Cochrane Collaboration's tool for assessing risk of bias in randomised trials. BMJ 2011;343:d5928-d5928.

15. Jonathan AC Sterne MAH, Barnaby C Reeves, Jelena Savović, Nancy D Berkman, Meera Viswanathan, David Henry, Douglas G Altman,, Mohammed T Ansari IB, James Carpenter, An-Wen Chan, Rachel Churchill, Asbjørn Hróbjartsson, Jamie Kirkham, Peter Jüni, Yoon Loke, Terri Pigott, Craig, Ramsay DR, Hannah Rothstein, Lakhbir Sandhu, Pasqualina Santaguida, Holger J Schünemann, Beverly Shea, Ian Shrier, Peter Tugwell, Lucy Turner, Jeffrey C, Valentine HW, Elizabeth Waters, Penny Whiting and Julian PT Higgins. The Risk Of Bias In Non-randomized Studies – of Interventions (ROBINS-I) assessment tool. Creative Commons Attribution-NonCommercial-NoDerivatives 4.0 International License., 2016.
